# PD-L1 negatively regulates antifungal immunity by inhibiting neutrophil release from bone marrow

**DOI:** 10.1038/s41467-022-34722-7

**Published:** 2022-11-11

**Authors:** Yao Yu, Rong-Rong Wang, Nai-Jun Miao, Jia-Jie Tang, Yun-Wei Zhang, Xiang-Ran Lu, Pei-Yi Yan, Jing Wang, Xin-Ming Jia

**Affiliations:** 1 Clinical Medicine Scientific and Technical Innovation Center, Shanghai Tenth People’s Hospital, Tongji University School of Medicine, 200092 Shanghai, China; 2 Shanghai Institute of Immunology, Department of Immunology and Microbiology, Shanghai JiaoTong University School of Medicine, 200025 Shanghai, China; 3 Department of Clinical Laboratory, Shanghai People’s Hospital of Putuo District, 200060 Shanghai, China

**Keywords:** Fungal infection, Neutrophils, Fungal host response

## Abstract

Programmed death ligand 1 (PD-L1) has been shown to be inducibly expressed on neutrophils to suppress host immunity during polymicrobial sepsis, virus and parasite infections. However, the role of PD-L1 on neutrophil-mediated antifungal immunity remains wholly unknown. Here, we show that the expression of PD-L1 on murine and human neutrophils was upregulated upon the engagement of C-type lectin receptor Dectin-1 with its ligand β-glucans, exposed on fungal pathogen *Candida albicans* yeast. Moreover, β-glucan stimulation induced PD-L1 translocation into nucleus to regulate the production of chemokines CXCL1 and CXCL2, which control neutrophil mobilization. Importantly, *C. albicans* infection-induced expression of PD-L1 leads to neutrophil accumulation in bone marrow, through mediating their autocrine secretion of CXCL1/2. Furthermore, neutrophil-specific deficiency of PD-L1 impaired CXCL1/2 secretion, which promoted neutrophil migration from bone marrow into the peripheral circulation, thereby conferring host resistance to *C. albicans* infection. Finally, either PD-L1 blockade or pharmacological inhibition of PD-L1 expression significantly increased neutrophil release from bone marrow to enhance host antifungal immunity. Our data together indicate that activation of Dectin-1/PD-L1 cascade by β-glucans inhibits neutrophil release from bone marrow reserve, contributing to the negative regulation of antifungal innate immunity, which functions as a potent immunotherapeutic target against life-threatening fungi infections.

## Abstract (web summary)

Programmed death ligand 1 (PD-L1) suppresses host immunity during infection and microbial sepsis. Here, the authors show that PD-L1 does this by regulating the secretion of CXCL1 and CXCL2 to control neutrophil mobilization during *Candida albicans* infection.

## Introduction

*Candida albicans* is both a commensal and opportunistic fungal pathogen of humans. During systemic infection, *C. albicans* enters the bloodstream and disseminates throughout the body, causing the disease known as invasive candidiasis. The successful clearance of *C. albicans* from the host mainly relies on neutrophils by releasing proinflammatory cytokines, producing reactive oxygen species (ROS) and anti-microbial peptides, and forming neutrophil extracellular traps (NETs)^1–3^. In addition, persistent neutropenia has been reported to be associated with an increased risk of developing invasive candidiasis^4,5^.

Neutrophil homeostasis in the peripheral circulation is tightly regulated and maintained by balancing neutrophil production in the bone marrow, the release from the bone marrow, and the clearance from the circulation. Mature neutrophils are retained in the bone marrow reserve and continuously released into the circulation under physiological conditions^6,7^. In response to various infections, neutrophil release from bone marrow reserve is rapidly increased and this process is mediated by the coordinated actions of granulocyte colony-stimulating factor (G-CSF), CXC chemokines KC (CXCL1), and macrophage inflammatory protein (MIP)−2 (CXCL2)^8,9^. Moreover, increased serum levels of CXCL1 and CXCL2, as potent chemo-attractants for neutrophils mobilization via their sole receptor CXCR2, can recruit neutrophils from peripheral circulation to the focus of infection. The regulation of neutrophil release from the bone marrow into the peripheral circulation is a critical step in their trafficking to inflammatory sites to maintain host protection from *C. albicans* infections.

Programmed death ligand 1 (PD-L1, encoded by *Cd274* gene) has generally been considered as a type I transmembrane protein that can interact with its receptor, programmed cell death 1 (PD-1, encoded by *Cd279* gene), thus inducing T cell de-activation and immune escape. PD-L1 is found to be expressed on the plasma membrane of immune cells, including neutrophils, B cells, dendritic cells (DCs), and macrophages^10,11^. During poly microbial sepsis, PD-L1 expression on neutrophils increases with inflammation and correlates with impaired antibacterial function^12,13^. Targeting PD-L1 with blocking antibodies can also enhance neutrophil innate immunity against bacterial infection^14,15^. Also, neutrophil PD-L1 has been shown to be induced directly by HIV-1 virus, bacterial lipopolysaccharide (LPS) and *Leishmania* parasite to interact with PD-1 on T cells, resulting in the persistent T cell exhaustion and immune suppression^16,17^. During *C. albicans* infection, it has been found that PD-L1 was inducibly expressed on T cells and natural killer cells, and immunotherapy with anti-PD-L1 antibody could abolish sepsis-induced immunosuppression and improve survival after bloodstream infection with *C. albicans*^18–20^. However, it remains wholly unknown about the effects of PD-L1 on the neutrophil-mediated antifungal immunity against *C. albicans* infection.

During *C. albicans* infection, β-glucans exposed on the cell wall of *C. albicans* play an important role in modulation of the host response. The C-type lectin receptor (CLR) Dectin-1 (encoded by *Clec7a* gene) is the most important neutrophil pattern recognition receptor (PRR) for the recognition of β-glucans^21,22^. In DCs and macrophages, the activation of Dectin-1 by β-glucans induces the production of the pro-inflammatory cytokines IL-12, IL-6 and IL-1β to drive Th1 and Th17-mediated protective responses against fungi infections^23^. In murine neutrophils, the engagement of Dectin-1 with β-glucans leads to the activation of integrin Mac-1 (CR3), which is essential for localizing cytotoxic responses of circulating neutrophils to infected tissues^24^. While in human neutrophils, Dectin-1 mutation causes the loss of its function, but affects neither its binding nor killing of *C. albicans*^25^. Besides, caspase-recruitment domain family member 9 (CARD9) is a key adaptor for the orchestration of spleen tyrosine kinase (Syk)-coupled Dectin-1-mediated antifungal immunity^26^. And of concern, it was reported that the neutrophils from patients with deficiency of CARD9 were impaired in the killing of un-opsonized *C. albicans*^27^. However, the role of β-glucans in the modulation of neutrophil-mediated antifungal immunity is poorly understood.

Here, we demonstrated that activation of Dectin-1 by fungal β-glucans induced PD-L1 expression in both murine and human neutrophils. Upregulated PD-L1 governed the chemotaxis of neutrophils through regulating their autocrine secretion of CXCL1 and CXCL2, which inhibited neutrophil release from the bone marrow into the peripheral circulation for aggravating *C. albicans* infection. These data together provide convincing evidence that PD-L1 negatively regulates host antifungal immunity against *C. albicans* infection through inhibiting neutrophil release from the bone marrow.

## Results

### β-glucans from *C. albicans* activate the Dectin-1/JAK2/STAT3 pathway to induce PD-L1 expression in neutrophils

**Fig. 1:**
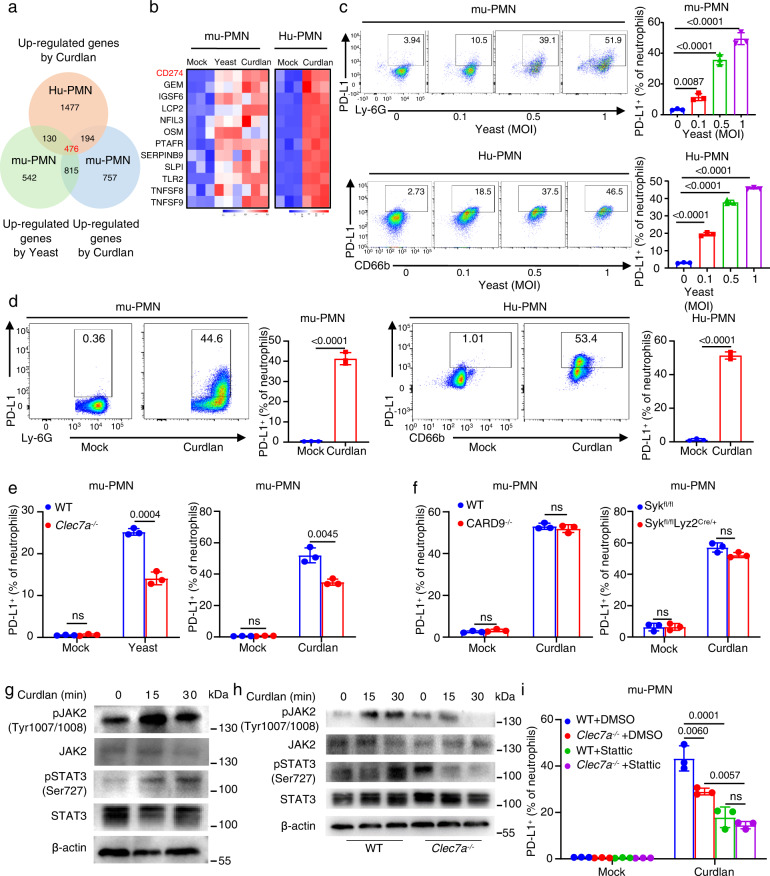
β-glucans from *C. albicans* activate the Dectin-1/JAK2/STAT3 axis to initiate PD-L1 expression in neutrophils. **a**, **b** RNA-seq analysis of bone marrow derived-neutrophils (mu-PMNs) and human neutrophils (Hu-PMNs), which were stimulated with curdlan (25 μg/well for mu-PMNs and 50 μg/well for Hu-PMNs) or heat-inactivated *C. albicans* yeast (MOI = 0.1) for 4 h. **a** Numbers of up-regulated genes as indicated. **b** Heatmaps of immune response-related genes of 476 co-upregulated genes as shown in **a**. **c** The percentage of PD-L1^+^Ly-6G^+^mu-PMNs and PD-L1^+^CD66b^+^Hu-PMNs after stimulation with heat-inactivated *C. albicans* yeast with indicated MOIs for 12 h. **d** The percentage of PD-L1^+^Ly-6G^+^mu-PMNs and PD-L1^+^CD66b^+^Hu-PMNs after stimulation with curdlan (25 μg/well for mu-PMNs and 50 μg/well for Hu-PMNs) for 12 h. **e** The percentage of PD-L1^+^Ly-6G^+^mu-PMNs from wild-type and *Clec7a*^−/−^ mice after stimulation with yeast (MOI = 1) or curdlan (25 μg/well) for 12 h. **f** The percentage of PD-L1^+^Ly-6G^+^mu-PMNs from wild-type, CARD9^−/−^ and Syk^fl/fl^Lyz2^Cre/+^ mice stimulated with curdlan(25 μg/well) for 12 h. **g** Immunoblotting analysis of phosphorylation of JAK2 or STAT3 in mu-PMNs stimulated with curdlan(25 μg/well) for the indicated time. **h** Immunoblotting analysis of phosphorylation level of JAK2 or STAT3 in wild-type and *Clec7a*^−/−^ mu-PMNs stimulated with curdlan(25 μg/well) for the indicated time. **i** The percentage of PD-L1^+^Ly-6G^+^mu-PMNs in wild-type and *Clec7a*^−/−^ mouse stimulated with curdlan (25 μg/well) combined with inhibitor Stattic (1 μΜ) for 12 h. Data were presented as mean ± SD; *n* = 3 (**c**–**f**, **i**) biological independent samples. Data were analyzed by unpaired two-sided Student’s *t* test in **d**–**f** or one-way ANOVA adjusted for multiple comparisons in **c**, **i**. Source data are provided as a Source data file.

To explore the β-glucan-induced common features of human and murine neutrophils, we performed the RNA-sequencing analysis and found that 476 up-regulated genes were overlapped in human and murine neutrophils after stimulation with β-glucan-containing particle curdlan or heat-inactivated *C. albicans* yeast (Fig. 1a and Supplementary Fig. 1a). Gene Ontology (GO) analysis revealed that these 476 up-regulated genes were highly relevant to inflammatory and immune responses (Supplementary Fig. 1b). Among these genes, the expression level of *Cd274* (encoding PD-L1), in murine and human neutrophils were significantly increased (Fig. 1b), which was verified by quantitative real-time PCR (Supplementary Fig. 1c). Furthermore, we used flow cytometry to validate that the frequency of human and murine neutrophils expressing PD-L1 was significantly increased after stimulation with heat-inactivated *C. albicans* yeast in a dose-dependent manner (Fig. 1c). Consistently, β-glucan stimulation also significantly increased the frequency of human and murine neutrophils expressing PD-L1 (Fig. 1d). Moreover, either deficiency of Dectin-1 in murine neutrophils or blockade of Dectin-1 in human neutrophils significantly decreased the PD-L1-expressing frequency induced by *C. albicans* yeast and β-glucans (Fig. 1e and Supplementary Fig. 1d, e). However, either the deficiency of Syk or CARD9, which are two well-characterized signal transducers of Dectin-1 downstream^26^, had no influences on the β-glucan-induced PD-L1-expressing frequency in murine neutrophils (Fig. 1f and Supplementary Fig. 1f). Together, these data indicated that β-glucans exposure at the surface of *C. albicans* yeast induced PD-L1 expression in human and murine neutrophils through Dectin-1, but not Syk and CARD9.

To explore the underlying mechanisms that Dectin-1 regulates PD-L1 expression in neutrophils, we further performed RNA-sequencing analysis and found that 54 up-regulated genes in murine neutrophils after stimulation with β-glucans or heat-inactivated *C. albicans* yeast were down-regulated due to the deficiency of Dectin-1 (Supplementary Fig. 1g). Kyoto Encyclopedia of Genes and Genomes (KEGG) analysis revealed that CLR pathway, Janus kinase (JAK) and signal transducer and activator of transcription (STAT) pathway, NF-κB pathway and PI3K-Akt pathway might be involved in regulating *C. albicans* yeast and β-glucan-induced expression of PD-L1 (Supplementary Fig. 1h). Importantly, we noticed that the inhibition of STAT3 activation with its specific inhibitor stattic, rather than bortezomib (NF-κB inhibitors) and wortmannin (PI3K-Akt inhibitors), most significantly decreased the frequency of murine neutrophils expressing PD-L1 induced by β-glucans (Supplementary Fig. 1i). Moreover, immunoblot-based assay showed that β-glucans stimulation significantly promoted the phosphorylation of JAK2 and STAT3 in murine neutrophils (Fig. 1g). Furthermore, Dectin-1 deficiency impaired β-glucan-induced phosphorylation of JAK2 and STAT3 in murine neutrophils (Fig. 1h). However, stattic treatment further decreased β-glucan-induced PD-L1-expressing frequency in Dectin-1-deficient murine neutrophils (Fig. 1i and Supplementary Fig. 1j), indicating that PD-L1 expression was mainly governed by JAK2/STAT3 pathway, which was partially regulated by Dectin-1. Thus, these data implied that the activation of Dectin-1 by β-glucans mediated the phosphorylation of JAK2 and STAT3 to regulate PD-L1 expression in neutrophils.

### PD-L1 governs the mobilization of neutrophils through regulating their autocrine secretion of CXCL1 and CXCL2

**Fig. 2:**
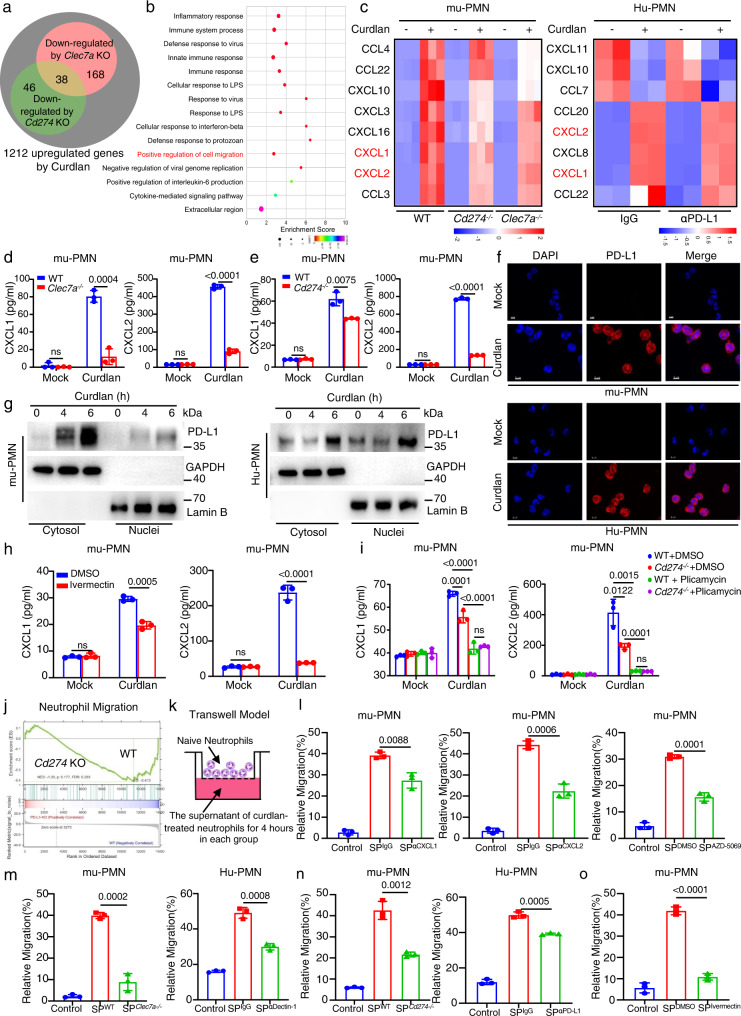
PD-L1 governs the mobilization of neutrophils through regulating their autocrine secretion of CXCL1/2. **a** Number of up-regulated genes in wild-type mu-PMNs and down-regulated genes in *Clec7a*^−/−^ and *Cd274*^−/−^ mu-PMNs stimulated with curdlan. **b** GO analysis of 38 down-regulated genes in *Clec7a*^−/−^ and *Cd274*^−/−^ mu-PMNs stimulated with curdlan. **c** Heatmaps show of down-regulated genes involved in positive regulation of cell migration as shown in **b** and in Hu-PMNs treated with anti-PD-L1(αPD-L1, 10 μg/ml), which were then stimulated with curdlan for 4 h. **d**, **e** ELISA quantification of CXCL1 and CXCL2 in the supernatant of *Clec7a*^−/−^ or *Cd274*^−/−^ mu-PMNs, which were stimulated with curdlan (25 μg/well) for 4 h. **f** Immunofluorescence staining of nuclear PD-L1 location in mu-PMNs stimulated with curdlan (25 μg/well) for 4 h and Hu-PMNs stimulated with curdlan (50 μg/well) for 6 h. Scale bar = 5 μm. **g** Representative histological images with immunoblotting assay of PD-L1 in mu-PMNs and Hu-PMNs, which were stimulated with curdlan for 6 h. **h** ELISA quantification of CXCL1 and CXCL2 in the supernatant of mu-PMNs, which were stimulated with curdlan (25 μg/well) and ivermectin (20 μM) for 4 h. **i** ELISA quantification of CXCL1 and CXCL2 in the supernatant of wild-type or *Cd274*^−/−^ mu-PMNs, which were stimulated with curdlan (25 μg/well) and inhibitor Plicamycin (5 nM) for 4 h. **j** Individual GSEA plots of neutrophil migration pathway in two independent RNA-Seq data of wild-type and *Cd274*^−/−^ mu-PMNs, which were stimulated with curdlan. *P* values were calculated by hypergeometric test and adjusted for multiple comparisons. **k** Schematic diagram of trans-well migration assay. The lower chamber contains the supernatant of neutrophils in different groups, which were stimulated with curdlan (25 μg/well for mu-PMNs and 50 μg/well for Hu-PMNs) for 4 h. **l**–**o** Trans-well migration assay of naive neutrophils driven by the supernatants of curdlan-stimulated wild-type, *Clec7a*^−/−^ or *Cd274*^−/−^ neutrophils combined with treatment of anti-CXCL1 (αCXCL1, 0.2 µg/ml), anti-CXCL2 (αCXCL2, 2 µg/ml), AZD-5069 (0.8 nM) or ivermectin (20 μM), and human neutrophils combined with treatment with anti-Dectin-1(αDectin-1, 1 μg/ml), anti-PD-L1(αPD-L1, 10 μg/ml). Data were presented as mean ± SD; *n* = 3 (**d**, **e**, **h**, **i**, **l**–**o**) biological independent samples. Data were analyzed by unpaired two-sided Student’s *t* test in **d**, **e**, **h**, **l**–**o** or one-way ANOVA adjusted for multiple comparisons in **i**. Source data are provided as a Source data file.

To explore the biological functions of PD-L1 in neutrophils, we performed RNA-sequencing analysis and found that 38 up-regulated genes in wild-type murine neutrophils after stimulation with β-glucans were down-regulated due to either the deficiency of Dectin-1 or PD-L1 (Fig. 2a and Supplementary Fig. 2a). Gene ontology (GO) analysis revealed that these 38 up-regulated genes were highly relevant to inflammatory and immune responses, and the positive regulation of cell migration (Fig. 2b). Among these up-regulated genes, the expression levels of genes encoding neutrophil chemoattractant CXCL1 and CXCL2 were decreased in either Dectin-1- or PD-L1-deficient murine neutrophils (Fig. 2c), which was verified by quantitative real-time PCR (Supplementary Fig. 2b). Direct ex vivo assay confirmed that β-glucan stimulation could induce the secretion of CXCL1 and CXCL2 in murine neutrophils (Supplementary Fig. 2c). In contrast, either the deficiency of Dectin-1 or PD-L1 in murine neutrophils significantly impaired β-glucan-induced secretion of CXCL1 and CXCL2 (Fig. 2d, e). We further found that the β-glucan-induced expression and secretion of CXCL1 and CXCL2 were significantly impaired in human neutrophils with blockade of Dectin-1 or PD-L1 compared with IgG control treatment using RNA-sequencing analysis (Supplementary Fig. 2d, e). Together, these data implied that PD-L1 could regulate the production of CXCL1 and CXCL2 in human and murine neutrophils.

We further explored the underlying mechanisms that PD-L1 regulates the β-glucan-induced production of CXCL1 and CXCL2 in human and murine neutrophils and found that PD-L1 was visualized to be overlapped with DAPI-labeling nucleus by immunofluorescence staining (Fig. 2f). Furthermore, we extracted nuclear proteins from human and murine neutrophils and found that, β-glucan stimulation induced a high level of PD-L1 translocation into nucleus (Fig. 2g). Moreover, blocking nuclear import of PD-L1 with ivermectin, which is a specific inhibitor of importin α/β-mediated nuclear import, could significantly inhibit β-glucan-induced production of CXCL1 and CXCL2 in murine neutrophils (Fig. 2h). These data indicated that β-glucan stimulation promoted PD-L1 translocation into the nucleus to regulate the production of CXCL1 and CXCL2 in neutrophils.

It has been shown that nuclear PD-L1 can couple to transcription factor Sp1 to regulate the synthesis of *Gas6* mRNA^28^. We found that the inhibition of Sp1 activation by plicamycin further decreased β-glucan-induced production of CXCL1 and CXCL2 in PD-L1-deficient murine neutrophils (Fig. 2i), indicating that CXCL1 and CXCL2 production was mainly controlled by Sp1 pathway, which might be partially regulated by PD-L1. Moreover, treatment with nuclear factor of activated T cells (NFAT) activation inhibitor in wild-type neutrophils resulted in comparable reductions in the amount of CXCL1 and CXCL2 compared with untreated PD-L1-deficient neutrophils, but this treatment further decreased CXCL1 and CXCL2 amount in PD-L1-deficient neutrophils (Supplementary Fig. 2f). These data implied that CXCL1 and CXCL2 production was also controlled by NFAT pathway, which might be partially regulated by PD-L1. However, inhibition of interferon regulatory factor 5 (IRF5) activation by N5-1 had no influences on β-glucan-induced production of CXCL1 and CXCL2 in either wild-type or PD-L1-deficient neutrophils (Supplementary Fig. 2g), implying that IRF5 was not involved in the regulation of CXCL1 and CXCL2 production.

It has been well characterized that CXCL1 and CXCL2, via their sole receptor CXCR2, are potent chemo-attractants for neutrophils. Gene set enrichment analysis showed that the deficiency of PD-L1 in neutrophils might affect their migration (Fig. 2j). We performed the trans-well migration assay that naive neutrophils were placed on the upper layer of a cell culture insert with permeable membrane and the supernatants of β-glucan-treated neutrophils with either treatment of blocking antibodies or the deficiency of Dectin-1 or PD-L1 were placed below the cell permeable membrane (Fig. 2k). We found that the supernatant of β-glucan-treated murine neutrophils for 4 h dramatically promoted neutrophil migration through the membrane (Fig. 2l and Supplementary Fig. 2h). However, the blockade of either CXCL1 or CXCL2 with their specific antibodies or treatment with AZD-5069, a specific antagonist of CXCR2, could significantly impair supernatant-induced neutrophil migration (Fig. 2l and Supplementary Fig. 2h). Moreover, the supernatant of β-glucan-treated murine and human neutrophils with either the deficiency or the blockade of Dectin-1 and PD-L1 failed to trigger neutrophil migration (Fig. 2m, n and Supplementary Fig. 2i, j). And the inhibition of PD-L1 translocation into the nucleus by ivermectin could also impair their supernatant-induced neutrophil migration (Fig. 2o and Supplementary Fig. 2k). Together, these data implied that PD-L1 governed the mobilization of neutrophils through regulating the autocrine secretion of CXCL1 and CXCL2.

### Bloodstream infection with *C. albicans* induces PD-L1 expression in neutrophils through Dectin-1 and subsequent neutrophil accumulation in the bone marrow

**Fig. 3:**
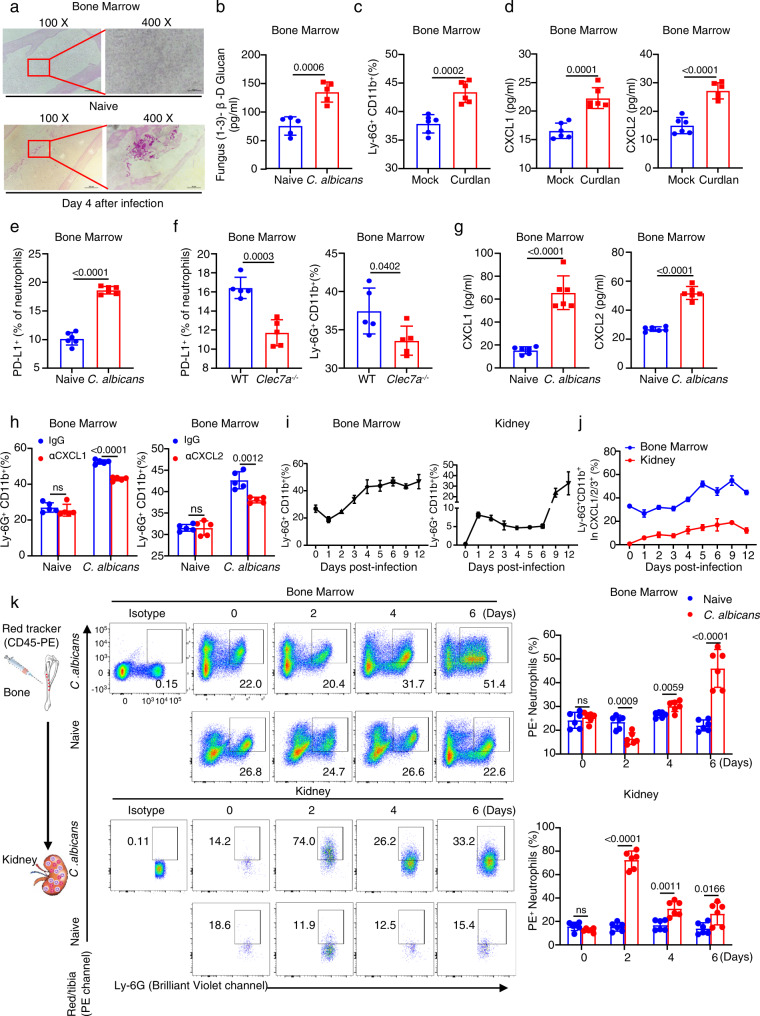
Bloodstream infection with *C. albicans* induces PD-L1 expression in neutrophils through Dectin-1 and subsequent neutrophil accumulation in the bone marrow. **a**, **b** Representative histological images with Periodic Acid-Schiff (PAS) staining (**a**) and G-test of β-glucans (**b**) in bone marrow of wild-type mice, which were intravenously infected with 2 × 10^5^ CFUs of *C. albicans* strain SC5314 for 4 days. **c**, **d** The percentage of neutrophils (**c**) and ELISA quantification of CXCL1 and CXCL2 (**d**) in the bone marrow of wild-type mice, which were microinjected with curdlan (0.1 μg dissolved in 0.1 mol/L NaOH) into the tibia for 2 days. **e** The percentage of PD-L1^+^Ly-6G^+^ neutrophils in the bone marrow of wild-type mice, which were intravenously infected with 2 × 10^5^ CFUs of *C. albicans* strain SC5314 for 4 days. **f** The percentage of PD-L1^+^Ly-6G^+^ (Left) and Ly-6G^+^ neutrophils (Right) in the bone marrow of wild-type and *Clec7a*^−/−^ mice, which were intravenously infected with 2 × 10^5^ CFUs of *C. albicans* strain SC5314 for 4 days. **g** ELISA quantification of CXCL1 and CXCL2 in the bone marrow of wild-type mice after intravenous infection with *C. albicans* SC5314 (2 × 10^5^ CFU/mouse) for 4 days. **h** The percentage of neutrophils in the bone marrow of naive and *C. albicans* (2 × 10^5^ CFU/mouse)-infected wild-type mice on Day 4, which were pretreated with IgG (Control, 5 or 40 ng/mouse), anti-CXCL1 (αCXCL1, 5 ng/mouse) and anti-CXCL2 (αCXCL2, 40 ng/mouse) into tibia for 24 h before scarification. **i** The percentage of neutrophils in the bone marrow and kidney of wild-type mice after intravenous infection with *C. albicans* SC5314 (2 × 10^5^ CFU/mouse) for indicated days. **j** The percentage of Ly-6G^+^ neutrophils in CXCL1/2/3^+^ cells in the bone marrow and kidney of *C. albicans* (2 × 10^5^ CFU/mouse)-infected wild-type mice for indicated days. **k** Neutrophil tracking assay in the bone marrow and kidney of wild-type mice, which were intravenously infection with *C. albicans* SC5314 (2 × 10^5^ CFU/mouse) for indicated days. PE-labeled anti-CD45 (10 μl) were microinjected into one tibia for 12 h before scarification at each indicated day. Data were presented as mean ± SD; *n* = 4 (**i**), *n* = 5 (**b**, **f**, **h**, **j**), *n* = 6 (**c**–**e**, **g**, **k**) biological independent samples. Data were analyzed by unpaired two-sided Student’s *t* test in **b**–**h**, **k**. Source data are provided as a Source data file.

We further explored the in vivo proportion variations of Ly6G^+^ neutrophils expressing PD-L1 in the bone marrow of mice after bloodstream infection with *C. albicans*. First, we performed histological analysis and found that fungi were visible in the bone marrow of infected mice with *C. albicans* on Day 4 (Fig. 3a). Meanwhile, quantitative assay by G-test showed that soluble β-glucans were significantly increased in the bone marrow of infected mice (Fig. 3b). Furthermore, the microinjection of β-glucans into the tibia significantly increased the frequency of Ly6G^+^ neutrophils and the amount of CXCL1 and CXCL2 in the bone marrow than that of naive mice (Fig. 3c, d and Supplementary Fig. 3a). Moreover, we found that *C. albicans* infection induced a greater frequency of Ly6G^+^ neutrophils expressing PD-L1 in the bone marrow than that of naive mice (Fig. 3e and Supplementary Fig. 3b). Importantly, Dectin-1 deficiency significantly impaired *C. albicans* infection-induced increase of neutrophil PD-L1 frequency and accumulation in the bone marrow (Fig. 3f and Supplementary Fig. 3c). Moreover, *C. albicans* infection induced a greater amount of CXCL1 and CXCL2 in the bone marrow of mice than that of naive mice (Fig. 3g) and blockade of either CXCL1 or CXCL2 with their respective antibodies through microinjection into the tibia of mice significantly reduced *C. albicans* infection-induced accumulation of neutrophils in the bone marrow (Fig. 3h and Supplementary Fig. 3d). Consequently, *C. albicans* infection time-dependently led to neutrophil accumulation in the bone marrow and its reduction in the kidney of mice (Fig. 3i and Supplementary Fig. 3e), which was the organ most infiltrated by adoptive transferred GFP^+^ neutrophils in *C. albicans*-infected mice compared with those in naive mice (Supplementary Fig. 3f). We further found that CXCL1 and CXCL2 were prominently produced by neutrophils in the bone marrow of *C. albicans*-infected mice, whereas, in the kidneys of infected mice, the proportion of CXCL1 and CXCL2 secreted by neutrophils was very low (Fig. 3j and Supplementary Fig. 3g). Together, these data indicated that *C. albicans* infection induced PD-L1 expression in neutrophils to regulate their autocrine secretion of CXCL1 and CXCL2, which led to neutrophil accumulation in the bone marrow of mice.

We next applied the cell tracking approach as described previously^29^ to determine whether bloodstream infection with *C. albicans* could affect neutrophil migration from the bone marrow into the infection sites. In detail, we performed the microinjection of PE-labeled CD45 antibody as a cell tracker into the tibia of mice. Twelve hours after microinjections into the tibia, we examined the proportion variations of PE-labeled neutrophils harvested from the bone marrow and kidney (Fig. 3k). We found that, on Day 2 after *C. albicans* infection, the frequency and number of PE-labeled neutrophils derived from the bone marrow was significantly increased in the kidney of mice (Fig. 3k). However, on Day 4 and 6 after *C. albicans* infection, the frequency and number of PE-labeled neutrophils in the kidney were dramatically decreased compared with those on Day 2 (Fig. 3k). Meanwhile, the frequency and number of PE-labeled neutrophils were significantly increased in the bone marrow of the infected mice on Day 4 and 6 compared with those on Day 2 (Fig. 3k). Thus, these data implied that *C. albicans* infection induced neutrophil accumulation in the bone marrow through inhibiting neutrophil migration into the circulation.

### Deficiency of PD-L1 significantly increases survival of *C. albicans*-infected mice through promoting neutrophil migration from the bone marrow into the kidney

**Fig. 4:**
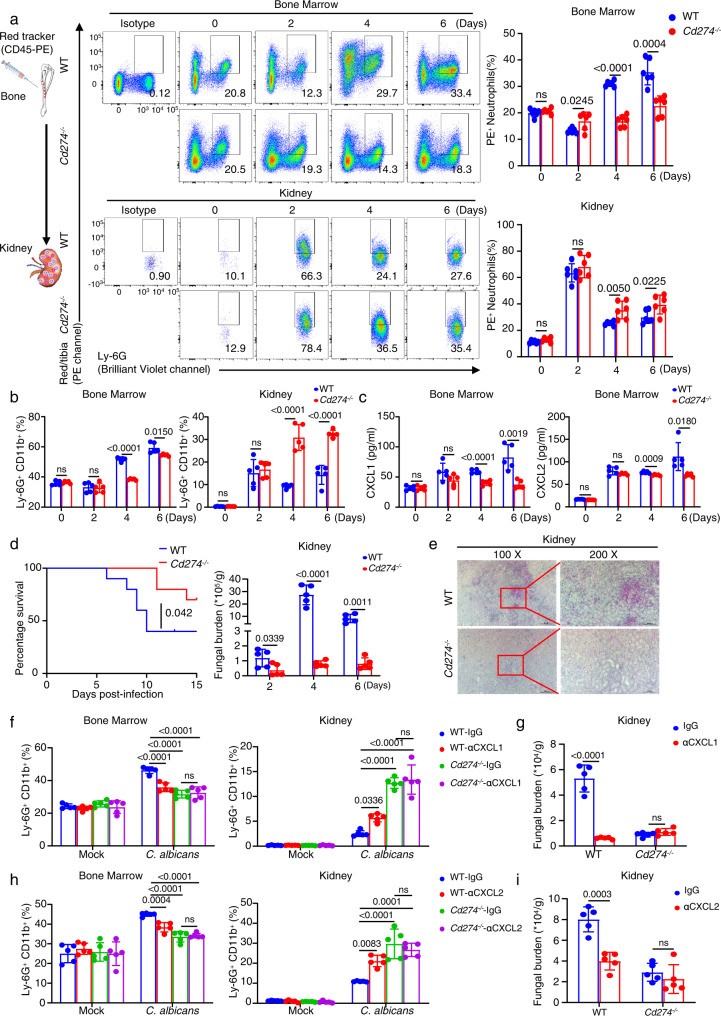
Deficiency of PD-L1 significantly increases survival of *C. albicans*-infected mice through promoting neutrophil migration from bone marrow into kidney. **a** Neutrophil tracking assay in the bone marrow and kidney of wild-type and *Cd274*^−/−^ mice, which were intravenously infection with *C. albicans* SC5314 (2 × 10^5^ CFU/mouse) for indicated days. PE-labeled anti-CD45 (10 μl) were microinjected into one tibia for 12 h before scarification at each indicated time. **b**–**e** The percentage of neutrophils in bone marrow and kidney (**b**), ELISA quantification of CXCL1 and CXCL2 in bone marrow (**c**), survival and kidney fungal burden (**d**), and PAS staining (**e**) of kidney of wild-type and *Cd274*^−/−^ mice, which were intravenously infection with *C. albicans* SC5314 (2 × 10^5^ CFU/mouse) for indicated days. Scar bars = 50 μm. **f**–**i** The percentage of Ly-6G^+^ neutrophils in bone marrow and kidney (**f**, **h**) and kidney fungal burden (**g**, **i**) of naive and *C. albicans* (2 × 10^5^ CFU/mouse)-infected wild-type and *Cd274*^−/−^ mice on day 4, which were pretreated with IgG (Control, 5 or 40 ng/mouse), anti-CXCL1 (αCXCL1, 5 ng/mouse), and anti-CXCL2 (αCXCL2, 40 ng/mouse) into tibia for 12 h before scarification. Data were presented as mean ± SD; *n* = 5 (**b**–**d**, **f**–**i**), *n* = 6 (**a**), *n* = 10 (**d**) biological independent samples. Data were analyzed by unpaired two-sided Student’s *t* test in **a**–**d**, **g**, **i**, one-way ANOVA adjusted for multiple comparisons in **f**, **h** or two-sided log rank (Mantel–Cox) tests in **d**. Source data are provided as a Source data file.

We further applied the cell tracking approach and found that the deficiency of PD-L1 significantly increased the frequency of PE-labeled neutrophil migration from the bone marrow into the kidney of mice on Day 4 and 6 after *C. albicans* infection (Fig. 4a). Meanwhile, the frequency of PE-labeled neutrophils was significantly decreased in the bone marrow of the infected mice on Day 4 and 6 (Fig. 4a). These data suggested that PD-L1 was involved in regulating neutrophil migration from the bone marrow into the kidney during *C. albicans* infection. Consequently, PD-L1 deficiency led to a significant decrease of neutrophil accumulation in the bone marrow and an obvious increase of neutrophil infiltration into the peripheral blood and kidney on Day 4 and 6 after *C. albicans* infection (Fig. 4b and Supplementary Fig. 4a, b). Importantly, PD-L1 deficiency significantly impaired the production of CXCL1 and CXCL2 in the bone marrow, but not peripheral blood and kidney, of mice on Day 4 after *C. albicans* infection (Fig. 4c and Supplementary Fig. 4c). However, the deficiency of PD-1 (encoded by *CD279* gene) had no effects on neutrophil accumulation in the bone marrow and kidney during *C. albicans* infection (Supplementary Fig. 4d). These data implied that PD-L1, independent on PD-1, governed the migration of neutrophils from the bone marrow into kidney through regulating their autocrine secretion of CXCL1 and CXCL2 during *C. albicans* infection.

We next examined the effect of PD-L1-mediated neutrophil migration on the host’s ability to clear systemic *C. albicans* infection and found that PD-L1 deficiency led to higher survival and lower fungal burden in the kidney than those in wild-type mice after infection with *C. albicans* (Fig. 4d). Periodic Acid-Schiff (PAS) staining of kidney displayed that the number of *C. albicans* was very few in the kidney of PD-L1-deficient mice compared with that in wild-type mice (Fig. 4e). Thus, these data indicated that PD-L1 inhibited neutrophil release from bone marrow into kidney to aggravate *C. albicans* infection.

To determine the roles of CXCL1 and CXCL2 in neutrophil migration from the bone marrow into kidney, we performed the microinjection of their respective neutralizing antibodies into the tibia of wild-type and PD-L1-deficient mice. We found that blockade of either CXCL1 or CXCL2 led to a significant decrease of neutrophil accumulation in the bone marrow and an obvious increase of neutrophil infiltration into kidney of wild-type mice on Day 4 after *C. albicans* infection, accompanied with a marked reduction of kidney fungal burden (Fig. 4f–i and Supplementary Fig. 4e, f). However, blocking either CXCL1 or CXCL2 had no influences on neutrophil migration from the bone marrow into kidney and fungal burden in PD-L1-deficient mice (Fig. 4f–i and Supplementary Fig. 4e, f), indicating that PD-L1 regulates the production of CXCL1 and CXCL2 to govern the neutrophil migration from the bone marrow into kidneys of *C. albicans*-infected mice.

### Neutrophil-specific deficiency of PD-L1 facilitates neutrophil migration from the bone marrow into the kidney of *C. albicans*-infected mice

**Fig. 5:**
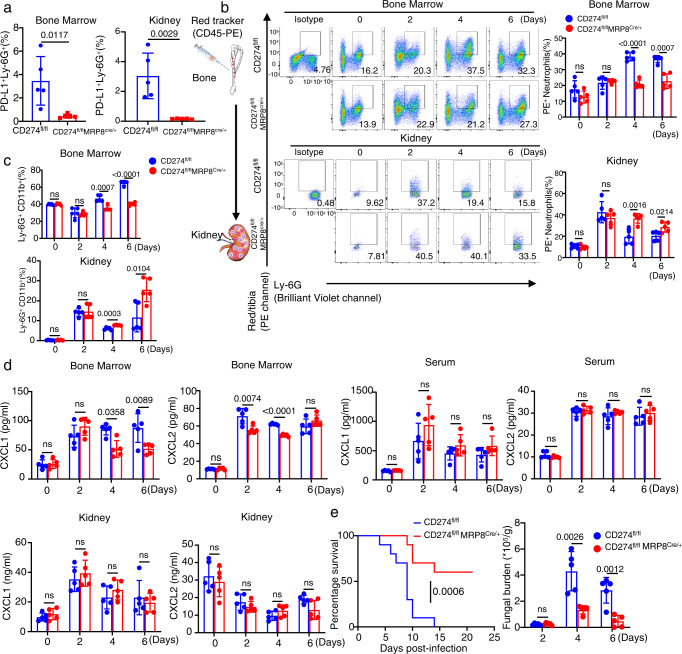
Neutrophil-specific deficiency of PD-L1 facilitates neutrophil migration from the bone marrow into the kidney of *C. albicans*-infected mice. **a** The percentage of PD-L1^+^ neutrophils in bone marrow and kidney of CD274^fl/fl^ and CD274^fl/fl^MRP8^Cre/+^ mice after infection with *C. albicans* SC5314 (2 × 10^5^ CFU/mouse) on Day 4. **b** Neutrophil tracking assay in the bone marrow and kidney of CD274^fl/fl^ and CD274^fl/fl^MRP8^Cre/+^ mice, which were intravenously infection with *C. albicans* SC5314 (2 × 10^5^ CFU/mouse) for indicated days. PE-labeled anti-CD45 (10 μl) were microinjected into one tibia for 12 h before scarification at each indicated time. **c**–**e** The percentage of neutrophils in bone marrow and kidney (**c**), ELISA quantification of CXCL1 and CXCL2 in bone marrow, serum and kidney (**d**), and survival and kidney fungal burden (**e**) of CD274^fl/fl^ and CD274^fl/fl^MRP8^Cre/+^ mice, which were intravenously infection with *C. albicans* SC5314 (2 × 10^5^ CFU/mouse) for the indicated days. Data were presented as mean ± SD; *n* = 5 (**a**–**e**), *n* = 10 (**e**) biological independent samples. Data were analyzed by unpaired two-sided Student’s *t* test in **a**–**e** or two-sided log rank (Mantel–Cox) tests in **e**. Source data are provided as a Source data file.

The original description of the MRP8^Cre^ transgene indicated high specificity in neutrophils^30^ and recent studies have also shown negligible deletion of floxed genomic sequences in MRP8^Cre^ transgene-positive macrophages or DCs^31,32^. To confirm the direct role of PD-L1 on neutrophil migration, we generated mice with conditional Cre/LoxP deletion of PD-L1 (CD274^fl/fl^MRP8^Cre/+^) in neutrophils by breeding CD274^fl/fl^ mice to MRP8^Cre/+^ mice. We confirmed that PD-L1 was deficient in Ly6G^+^ neutrophils in the bone marrow and kidney of infected mice with *C. albicans* (Fig. 5a and Supplementary Fig. 5a). We further applied the cell tracking approach and found that neutrophil-specific deficiency of PD-L1 significantly increased the frequency of PE-labeled neutrophil migration from the bone marrow into the kidneys of mice on Day 4 and 6 after *C. albicans* infection (Fig. 5b). Consequently, neutrophil-specific deficiency of PD-L1 led to a significant decrease of neutrophil accumulation in the bone marrow and an obvious increase of neutrophil infiltration into the peripheral blood and kidney on Day 4 and 6 after *C. albicans* infection (Fig. 5c and Supplementary Fig. 5b, c). Importantly, neutrophil-specific deficiency of PD-L1 significantly impaired the production of CXCL1 and CXCL2 in the bone marrow, but not peripheral blood and kidney, of mice on Day 4 and 6 after *C. albicans* infection (Fig. 5d). Moreover, neutrophil-specific deficiency of PD-L1 led to higher survival and lower fungal burden in the kidney than those in CD274^fl/fl^ mice after infection with *C. albicans* (Fig. 5e). Thus, these data confirmed that *C. albicans* infection induced PD-L1 expression in neutrophils, which inhibited neutrophil release from the bone marrow into the kidney to aggravate *C. albicans* infection.

### PD-L1 blockade facilitates neutrophil-based immunotherapy against lethal *C. albicans* sepsis

**Fig. 6:**
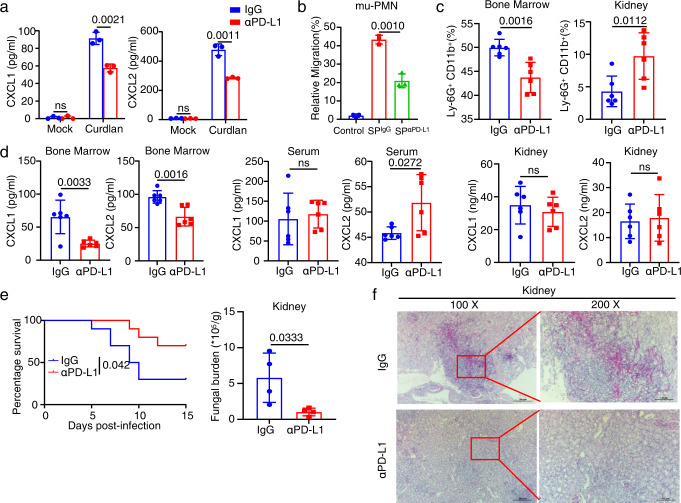
PD-L1 blockade facilitates neutrophil-based immunotherapy against lethal *C. albicans* sepsis. **a** ELISA quantification of CXCL1 and CXCL2 in the supernatant of wild-type mu-PMNs treated with anti-PD-L1 (αPD-L1, 10 μg/ml), which were co-stimulated with curdlan (25 μg/well) for 4 h. **b** Trans-well migration assay of naive neutrophils driven by the supernatants of wild-type mu-PMNs stimulated with curdlan (25 μg/well) combined with anti-PD-L1 (αPD-L1, 10 μg/ml) or IgG treatment for 4 h. **c**–**f** The percentage of neutrophils in bone marrow and kidney (**c**), ELISA quantification of CXCL1 and CXCL2 in bone marrow, serum and kidney (**d**), survival and fungal burden of kidney (**e**), and representative histological images with Periodic Acid-Schiff (PAS) staining of kidneys (**f**) of *C. albicans* (SC5314, 2 × 10^5^ CFU/mouse)-infected wild-type mice, which were treated by IgG (Control, 200 μg/mouse) and anti-PD-L1 (αPD-L1, 200 μg/mouse) for indicated days. Scar bars = 50 μm. Data were presented as mean ± SD; *n* = 3 (**a**, **b**), *n* = 4 (**e**), *n* = 6 (**c**, **d**), *n* = 10 (**e**) biological independent samples. Data were analyzed by unpaired two-sided Student’s *t* test in **a**–**e** or two-sided log rank (Mantel–Cox) tests in **e**. Source data are provided as a Source data file.

We further explored the effects of PD-L1 blockade using its specific antibody on the functions of neutrophils both ex vivo and in vivo. We found that PD-L1 blockade in murine neutrophils significantly inhibited β-glucan-induced secretion of CXCL1 and CXCL2 (Fig. 6a). Consequently, the supernatant of β-glucan-treated murine neutrophils with PD-L1 blockade failed to trigger neutrophil migration (Fig. 6b and Supplementary Fig. 6a). Moreover, mice receiving PD-L1 blocking antibody showed a significant decrease of neutrophil accumulation in the bone marrow and a significant increase of neutrophil infiltration into the kidney compared with those receiving isotype IgG treatment on Day 4 after bloodstream infection with *C. albicans* (Fig. 6c and Supplementary Fig. 6b).

Importantly, PD-L1 blockade significantly decreased the amount of CXCL1 and CXCL2 in the bone marrow, but not peripheral blood and kidney, of mice after *C. albicans* infection (Fig. 6d). And mice receiving PD-L1 blocking antibody presented higher survival and lower fungal burden in the kidney than those receiving isotype IgG treatment after infection with *C. albicans* (Fig. 6e). PAS staining of kidney also displayed that PD-L1 blockade revealed hardly any *C. albicans* in the kidney compared with receiving isotype IgG treatment in mice (Fig. 6f). Together, these data indicated that PD-L1 blockade could promote neutrophil migration from the bone marrow into the kidney for facilitating neutrophil-based immunotherapy against lethal *C. albicans* sepsis.

### ACT001 inhibits PD-L1 expression to enhance neutrophil-mediated antifungal immunity against lethal *C. albicans* sepsis

**Fig. 7:**
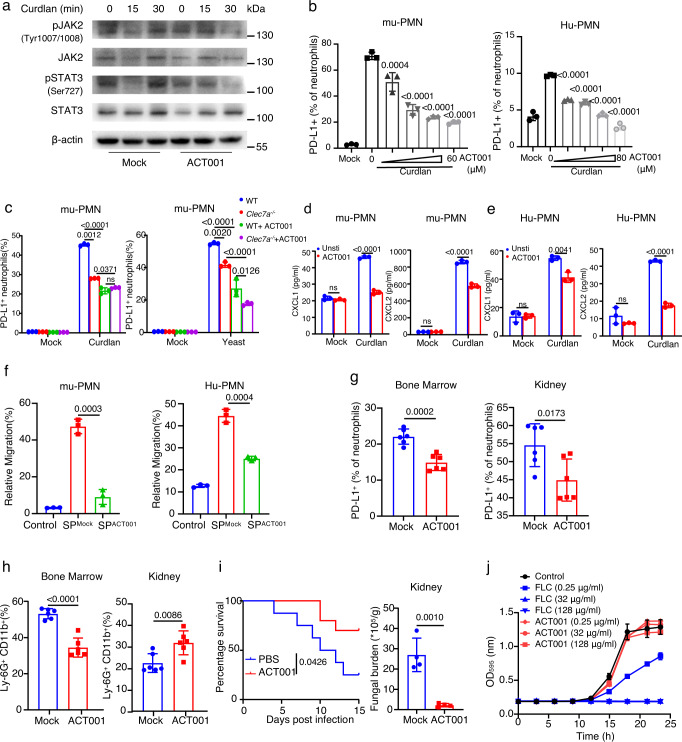
ACT001 inhibits PD-L1 expression to enhance neutrophil-mediated antifungal immunity against lethal *C. albicans* sepsis. **a** Immunoblotting analysis of phosphorylation level of JAK2 or STAT3 in mu-PMNs after treatment with ACT001 (40 μmol/L), which were co-stimulated with curdlan (25 μg/well) for indicated time. **b** The percentage of PD-L1^+^Ly-6G^+^mu-PMNs and PD-L1^+^CD66b^+^Hu-PMNs after treatment with ACT001 at indicated concentrations, which were co-stimulated with curdlan (25 μg/well for mu-PMNs and 50 μg/well for Hu-PMNs) for 12 h. **c** The percentage of PD-L1^+^ Ly-6G^+^ mu-PMNs in wild-type and *Clec7a*^−/−^ mouse stimulated with curdlan (25 μg/well) or yeast (MOI = 1) combined with inhibitor ACT001 (40 μmol/L) for 12 h. **d**, **e** ELISA quantification of CXCL1 and CXCL2 in the supernatant of mu-PMNs and Hu-PMNs after treatment with ACT001 (40 μmol/L), which were co-stimulated with curdlan (25 μg/well for mu-PMNs and 50 μg/well for Hu-PMNs) for 4 h. **f** Naive neutrophils driven by the supernatants of wild-type mu-PMNs stimulated with curdlan (25 μg/well for mu-PMNs and 50 μg/well for Hu-PMNs) combined with ACT001 (40 μmol/L) for 4 h. **g**–**i** The percentage of PD-L1^+^ neutrophils (**g**) and neutrophils (**h**), and survival and fungal burden of kidney (**i**) of *C. albicans* (2 × 10^5^ CFU/mouse)-infected wild-type mice, which were intragastrically treated with ACT001 (200 mg/kg) on day 1 and 3. **j** In vitro antifungal susceptibility testing results. Data were presented as mean ± SD; *n* = 3 (**b**–**f**), *n* = 4 (**i**), *n* = 6 (**g**, **h**), *n* = 10 (**i**) biological independent samples. Data were analyzed by unpaired two-sided Student’s *t* test in **d**–**i**, one-way ANOVA adjusted for multiple comparisons in **b**, **c** or two-sided log rank (Mantel–Cox) tests in **i**. Source data are provided as a Source data file.

It has been shown that ACT001, which is derived from the structural modification of a well-studied anti-inflammatory and anticancer agent parthenolide^33^, can block PD-L1 expression by inhibiting the phosphorylation of STAT3 in glioblastoma^34^. We found that ACT001 treatment could inhibit β-glucan-induced phosphorylation of JAK2 and STAT3 in murine neutrophils (Fig. 7a). Moreover, ACT001 treatment significantly inhibited β-glucan-induced expression of PD-L1 in murine and human neutrophils in a dose-dependent manner (Fig. 7b). However, ACT001 treatment further decreased β-glucan or *C. albicans* yeast-induced PD-L1-expressing frequency in Dectin-1-deficient murine neutrophils (Fig. 7c and Supplementary Fig. 7a), which further confirmed that PD-L1 expression was mainly governed by STAT3 pathway, which was partially regulated by Dectin-1. Moreover, ACT001 treatment significantly inhibited β-glucan-induced secretion of CXCL1 and CXCL2 by murine and human neutrophils (Fig. 7d, e). Consequently, the supernatant of β-glucan-treated murine neutrophils combined with ACT001 treatment failed to trigger the migration of murine and human neutrophils (Fig. 7f and Supplementary Fig. 7b). Importantly, ACT001 treatment significantly reduced the frequency of Ly6G^+^ neutrophils expressing PD-L1 in both bone marrow and kidney of infected mice with *C. albicans* (Fig. 7g and Supplementary Fig. 7c). Moreover, ACT001 treatment led to a significant decrease of neutrophil accumulation in the bone marrow and a significant increase of neutrophil infiltration into the kidney of mice on Day 4 after *C. albicans* infection (Fig. 7h and Supplementary Fig. 7d). Also, ACT001 treatment led to higher survival and lower fungal burden in the kidney of infected mice (Fig. 7i). Furthermore, ACT001 had no killing effects on *C. albicans* by in vitro antifungal susceptibility testing (Fig. 7j). Thus, these data implied that ACT001 might be a promising drug for the treatment of systemic *C. albicans* infection by inhibiting PD-L1 expression to enhance neutrophil-mediated antifungal immunity.

## Discussion

Among the different types of PRRs, Dectin-1 has been universally regarded as the principal receptor for β-glucans. In DCs and macrophages, Dectin-1 signaling activated by fungi-derived β-glucans is initiated by phosphorylation of Syk, which is orchestrated for CARD9-mediated canonical signaling via the transcription factor NF-κB subunit p65 and the kinase ERK^26,35^. However, it remains largely unknown about Dectin-1-mediated signaling pathways in neutrophils. Recently, it has been shown that in human neutrophils, Dectin-1 is dispensable for their killing of *C. albicans*, but Syk and CARD9 play the role^25^. Apart from that, in our present study, we show that in murine and human neutrophils, activation of Dectin-1 by β-glucans or *C. albicans* yeast induces PD-L1 expression through mediating the downstream activation of JAK2/STAT3 cascade which has never been determined before. Thus, that is to say, in murine and human neutrophils, β-glucan-induced PD-L1 expression can significantly be blocked by either the deficiency or blockade of Dectin-1 through impairing the phosphorylation of JAK2 and STAT3. Moreover, inhibition of STAT3 activation by its inhibitor also significantly blocks β-glucan-induced expression of PD-L1 in murine neutrophils. Coincidentally, it has also been shown that tumor-derived GM-CSF, lactate, and extracellular vesicles can induce PD-L1 expression in neutrophils through activating JAK and STAT3 signaling pathway^36–38^. However, either the deficiency of Syk or CARD9 has no effects on β-glucan-induced PD-L1 expression in murine neutrophils. Together, our data provide convincing evidence that the engagement of Dectin-1 with its ligand fungi-derived β-glucans is essential for regulating PD-L1 expression in neutrophils through activating the JAK2/STAT3 cascade, while both Syk and CARD9 are dispensable for this process.

Recent evidence has suggested that PD-L1 expression in neutrophils, which is associated with suppressor capacity to interact with PD-1 on T cells, plays an important role in numerous diseases, including acquired immune deficiency syndrome (AIDS)^17^, sepsis^39^, *Burkholderia pseudomallei*-infected disease^40^, tuberculosis^41^, *Leishmania* parasite infection^16^, systemic lupus erythematosus^42^, and injury-induced infection^15^. Here, in the present study, we show that during *C. albicans* infection, PD-L1 governs the mobilization of neutrophils through regulating their autocrine secretion of CXCL1 and CXCL2. In support of the above mechanism, we present that deficiency or blockade of PD-L1 significantly impairs the secretion of CXCL1 and CXCL2 in murine or human neutrophils induced by β-glucans, and subsequently suppresses neutrophil mobilization. For further confirming the pathophysiological relevance of such in vitro findings, we demonstrate that PD-L1, independent of PD-1, governs mobilization of neutrophils from the bone marrow reserve into the kidney through regulating their autocrine secretion of CXCL1 and CXCL2, during bloodstream infection with *C. albicans*. Thus, in other words, neutrophil-specific deficiency of PD-L1 during *C. albicans* infection results in a significant decrease in accumulation or deposits of neutrophils in the bone marrow, by impairing the production of CXCL1 and CXCL2, thereby more likely to migrate to the most infected foci during *C. albicans* infection to exert the effect. Coincidentally, recent studies have shown that, PD-L1 expression in DCs induced by α-(1,3)-glucan from *Aspergillus fumigatus* through activating Wnt/β-catenin pathway promotes regulatory T-cell (Treg) polarization, suggesting that PD-L1 in DCs negatively regulates α-(1,3)-glucan-induced protective immune responses against *A. fumigatus* infection^43,44^. Moreover, it has been further reported that, PD-L1 expression in macrophages induced by *Cryptococcus neoformans* infection suppresses macrophage activation, and PD-L1 expression in T cells induced by *C. albicans* infection can promote T cell exhaustion^19,45^. It remains unclear whether PD-L1 expression in neutrophils induced by β-glucans can affect Treg polarization, macrophage activation or T cell exhaustion during *C. albicans* infection, which would be further explored in our future studies.

More notably, we found that PD-L1 deficiency remarkably improves the survival of *C. albicans*-infected mice. This might provide a new target for enhancing neutrophil-mediated anti-fungal immunity regulated by PD-L1. It has been reported that, in the treatment of severe sepsis caused by fungi, granulocyte transfusion and administration of G-CSF are used for antifungal therapy as adjunctive therapies combined with antifungal agents, simply by increasing the number of neutrophils^46,47^, while these therapies have been considered to produce serious adverse effects^46,48,49^. However, in our study, during *C. albicans* infection, we show that blockade or pharmacological inhibition of PD-L1 expression promotes neutrophil release from bone marrow reserves to the site of inflammation, more precisely regulating the direction of neutrophil migration. Targeting PD-L1 might be critical for developing neutrophil-based immunotherapy involved against fungal infections. These data together provide persuasive evidence that PD-L1 negatively regulates host antifungal immunity against *C. albicans* infection through inhibiting neutrophil release from bone marrow.

Accumulating evidence suggests that neutrophils can secrete CXCL1 and CXCL2 in an autocrine manner when stimulation with multiple stimulants including LPS, TNF-α, IFN-γ and G-CSF^50^. Moreover, recent study has revealed that CXCL1 is mainly produced by macrophages and epithelial cells whereas CXCL2 is almost exclusively derived from neutrophils^51^. In the present study, we show that β-glucan stimulation induced the secretion of CXCL1 and CXCL2 in human and murine neutrophils whereas PD-L1 deficiency in neutrophils inhibited the secretion of CXCL1 and CXCL2 induced by β-glucans. These data implied that PD-L1 regulated β-glucan-induced CXCL1 and CXCL2 production of neutrophils. It has been shown that CXCL1 and CXCL2 have highly homologous structures with about 90% in amino acid sequence and bind to their sole receptor CXCR2^51^. However, the mechanisms underlying PD-L1 regulates the expression of CXCL1 and CXCL2 in neutrophils remain largely unknown. As to this problem, we demonstrate that PD-L1 translocated into the nucleus to regulate the production of CXCL1 and CXCL2 in neutrophils. In addition, it has been described that nuclear PD-L1 can couple with the transcription factor Sp1, which is involved in the regulation of CXCL1 expression^28^. Further studies are required to elucidate the interaction of nuclear PD-L1 with Sp1 to regulate the expression of CXCL1 and CXCL2 in neutrophils.

In summary, our data suggest that activation of Dectin-1 by fungal β-glucans induced PD-L1 expression in murine and human neutrophils, and upregulated PD-L1 governs the mobilization of neutrophils through regulating their autocrine secretion of CXCL1 and CXCL2. Our work uncovers a negative role of PD-L1 expression on the host immune response, which inhibits neutrophil migration from the bone marrow into the infected sites for favoring immune escape of fungi infections. It provides new insights that PD-L1 may function as a potent therapeutic target of neutrophil-based immunotherapy against fungal infections through regulating neutrophil release from the bone marrow. Understanding the role of PD-L1 in neutrophil-mediated antifungal immunity will open up a new avenue for developing immunotherapy approaches against various infectious diseases.

## Methods

### Ethics statement

All animal experiments were performed according to the protocol approved by the Animal Ethics Committee of Tongji University School of Medicine (protocol No. TJAA09021102). Studies enrolling human neutrophils were approved by the Human Research Committee of Tongji University School of Medicine (protocol No. 2021TJDX018).

### Mice

C57BL/6 mice were purchased from Gempharmatech Co., Ltd (Nanjing, China). Dectin-1 and CARD9 knockout (KO) mice were from X. Lin (Tsinghua University School of Medicine, Beijing, China). *Cd274* KO mice were from L. Zhou (NHFPC Key Laboratory of Combined Multi-organ Transplantation, Hangzhou, China). *Cd279* KO mice were from Y. Yu (Tongji University, Shanghai, China). Syk^fl/fl^Lyz2^Cre/+^ mice were from H. Xiao (Institute Pasteur of Shanghai, China). CD274^fl/fl^ and MRP8^Cre/+^ mice were purchased from GemPharmatech. All mice were genotyped by PCR using genomic tail DNA. All animal experiments were undertaken in accordance with the National Institutes of Health Guide for the Care and Use of Laboratory Animals. All mice were housed in Tongji University specific pathogen free (SPF) animal facility. Housing conditions were as following: dark/light cycle 12/12 h, ambient temperature around 21–22 °C, and humidity between 40 and 70%.

### Human subjects

The human neutrophils were isolated from peripheral blood of 18–60-year-old healthy control individuals (*n* = 15). Participants were recruited via advertising on school web-pages and suggested by clinical doctors. The purity and activity (>95%) of human neutrophils were confirmed by flow cytometry. All participants were informed both orally and in writing of potential risks, and discomforts associated with participation before written consent was obtained.

### Neutrophil preparation

Neutrophils were isolated as previously described^52,53^. Murine bone marrow was isolated from femur and tibia by flushing with the sorting buffer (1 × HBSS with 1%FBS). Bone marrow-derived neutrophils were obtained by centrifugation with a discontinuous Percoll (GE Healthcare) gradient (75, 66, and 52%) and harvested between the 75 and 66% layers. The remaining erythrocytes were removed through hypotonic lysis by ddH_2_O.

Human neutrophils were purified from venous blood of healthy volunteers by the classical 2-step method. The erythrocytes were removed through 3% dextran (Sigma) sedimentation and then peripheral blood mononuclear cells were removed by Ficoll-Paque (GE Healthcare) density centrifugation. The remaining erythrocytes were removed through hypotonic lysis by ddH_2_O.

The sorted neutrophils are confirmed for their purity and activity (>95%) by FCM. Neutrophils were suspended in RPMI1640 medium with 10% heated inactivated FBS and 1% Penicillin/Streptomycin at 1.0 × 10^7^ cells/ml.

### *C. albicans* strains and culture condition

*C. albicans* strain SC5314 were purchased from ATCC (American Type Culture Collection). *C. albicans* yeast cells were grown in YPD (yeast extract, peptone, and dextrose)-rich medium at 30 °C for 16 h.

### Bloodstream infection murine model

*C. albicans* SC5314 induced infectious model was established as previously described^54^. In brief, six-to-eight-week-old female or male mice were intravenously injected with *C. albicans* SC5314 (2 × 10^5^ CFU) in 200 μl PBS.

### RNA-seq analysis of neutrophils

Murine neutrophils were stimulated with curdlan (25 μg/well) or heat-inactivated *C. albicans* yeast (MOI = 0.1) for 4 h. Human neutrophils were stimulated with curdlan (50 μg/well) for 4 h. Neutrophils were collected and frozen in Lysis/Binding Buffer and total RNA extracted using miRVana kit (Invitrogen). The integrity of RNA was assessed using the Agilent 2100 Bioanalyzer (Agilent Technologies, Santa Clara). Then the libraries were constructed using TruSeq Stranded mRNA LT Sample Prep Kit (Illumina). The transcriptome sequencing and analysis were conducted by OE Biotech Co., Ltd. (Shanghai, China).

Differential expression analysis was performed using the DESeq (2012) R package. *T*-test threshold (p values <0.05) and fold-change threshold (>1.5 or <0.5) were set as the threshold for significantly differential expression genes (DEGs). GO enrichment and KEGG pathway enrichment analysis^55^ of DEGs were performed respectively using R based on the hypergeometric distribution. Heatmapping were performed in R package version1.0.8. Gene set enrichment analysis (GSEA 3.0, Broad Institute) was performed to determine gene sets and pathways that are significantly enriched in DEGs for each group of comparison against the GSEA molecular signature database.

### Flow cytometry

For surface staining, cells were washed and stained with the following fluorescent conjugated antibodies: PE Rat anti-Mouse CD274 antibody (BD Biosciences, 558091, 1:100); Brilliant Violet 421™ anti-mouse Ly-6G antibody (BioLegend, 127628, 1:100); PerCP/Cyanine5.5 anti-mouse/human CD11b antibody (BioLegend, 101228, 1:100); PE anti-mouse CD45 antibody (BioLegend, 103106, 1:100); BV421 Mouse anti-Human CD66 antibody (BD Biosciences, 562741, 1:100); eBioscience™ Fixable Viability Dye eFluor™ 780 (Invitrogen™, 103106, 1:1000) for 30 min at 4 °C. For intracellular protein staining, cells were collected for surface staining and then intracellular staining using the Transcription Factor Buffer Set (BD Pharmingen). After washing with PBS, the cells were run on a FACSCelesta (BD Biosciences) using BD DIVA8.0.1 software (BD Biosciences), and data were analyzed with Flowjo v10.

### Immunoblotting

Cells (1.0 × 10^7^) were lysed using Qproteome Mammalian Protein Prep Kit (Qiagen). Lysates were briefly sonicated, clarified, then subjected to SDS-PAGE and transferred to PVDF membranes (Merck Millipore) using a transfer apparatus (Bio-Rad). Primary antibodies which were used in this study: Phospho-Jak2 (C80C3, CST, 3776S, 1:1000); Phospho-Stat3 (Ser727, CST, 9134S, 1:1000); Jak2 (D2E12, CST, 3230S, 1:1000); Stat3 (D3Z2G, CST, 12640S, 1:1000); PD-L1 (EPR20529, Abcam, ab213480, 1:1000; Absin abs136046, 1:1000); β-Actin (8H10D10, CST, 3700S, 1:1000); GAPDH (D16H11, CST, 5174S, 1:1000); Lamin B1 (D4Q4Z, CST, 12586S, 1:1000). Primary antibody binding was visualized by chemiluminescence using HRP-conjugated goat anti-rabbit or goat anti-mouse IgG secondary antibodies (1:5000; Cell Signaling Technology).

### RNA purification, cDNA synthesis, and qPCR analysis

Total RNA was isolated using the RNeasy Mini Kit (Qiagen). cDNA was synthesized using PrimeScript™ RT Master Mix (Takara) following the manufacturer’s instructions. qPCR reactions were performed using 2 × SYBR Green qPCR Master Mix (Bimake) and a CFX96 Real-Time System instrument (Bio-Rad). Thermal cycling conditions included an initial denaturation step of 95 °C for 10 min and 40 cycles at 95 °C for 15 s and 60 °C for 60 s. Analyses were carried out in triplicate for each data point. The sequences of qPCR primers were listed in Table S1.

### Cytokine measurement

Neutrophils were stimulated with curdlan for 4 h. Then, the supernatants of neutrophils were collected. The protein levels of CXCL1 and CXCL2 were measured by enzyme-linked immunosorbent assay kits (R&D Systems, Invitrogen, MULTI SCIENCES) according to the manufacturer’s instructions.

### Immunofluorescence

Neutrophils were stimulated as indicated. In all, 1.0 × 10^6^ cells were seeded on 35 mm confocal dishes (Beyotime), which were coated with 0.001% poly-L-Lysine overnight. Cells were fixed in 4% paraformaldehyde for 30 min at 4 °C. Then the cells were washed with PBS only once, and permeabilized in 0.1% triton X-100 at 4 °C for 10 min. Cells were blocked with 5% BSA at room temperature for 1 h, followed by incubation with anti-PD-L1 antibody (Proteintech, 66248-1-Ig, 1:200) overnight at 4 °C. Cells were washed with PBS only once, and incubated with anti-rabbit IgG (Cell signaling technology, 4412 S, 1:200) for 1 h at room temperature. Cells were washed with PBS and then incubated with DAPI (1 μg/ml) for 15 min at room temperature. Images were captured with FV3000 confocal system (Olympus) and data were analyzed with Imaris (Bitplane, V9.5).

### Trans-well migration assay

Cell migration assay was carried out by using trans-well chambers with inserts of 5-μm pore size (Costar). In all, 4 × 10^5^ cells were resuspended in 200 μl 1640 medium with 10% FBS. Add 600 μl supernatant for cells of each experimental condition to the bottom of each well and 200 μl cell suspension to the top of each chamber. The cells were allowed to migrate for 2 h at 37 °C and 5% CO_2_. The chambers were then removed from the wells and the number of cells in the bottom of each well was counted using the automated cell counter (Countstar). The migrated cells were fixed with 4% paraformaldehyde and stained with crystal violet.

### Isolation of kidney immunocytes

Mice were euthanized and kidney were harvested. Tissues were carefully minced and digested with 1 mg/mL collagenase D (Roche) for 120 min. Digestion was quenched by RPMI1640 medium with 10% heated inactivated FBS, and filtered with 70 μm Nylon mesh to obtain cell suspensions. For enriching immunocytes in kidney, cells were centrifuged at 1500 × *g* for 45 min by using 40 and 80% Percoll gradients (GE Healthcare) and harvested cell layer between them. Kidney immunocytes were collected and used for flow cytometry analysis.

### Bone marrow tagging by microinjection

Bone marrow tagging by microinjection were performed as previously described^29^. Microinjection of the tibia marrow was performed sequentially under 0.3% pentobarbital anesthesia, using a 1 ml syringe mounted with a 5 mm-outer-diameter veterinary needle. For each procedure aseptic technique was used. For the tibia marrow injection, we injected at the chosen injection site of tibia plateau just below the knee and the bone wall was perforated with a veterinary needle. The red cell tracker or CXCL1/2 antibodies (Clone # 48415, R&D Systems, MAB453, 0.2 μg/ml or 5 ng/mice once; Clone # 40605, R&D Systems, MAB452, 2 μg/ml or 40 ng/mice once) was injected in the marrow cavity over 30 s. Animals were sacrificed 12 h after injection.

### Statistics and reproducibility

All experiments were performed at least three times. Data shown are representative of three biological replicates. Representative images of immunofluorescence staining, PAS staining, and western blot assays are shown. Data were analyzed using GraphPad Prism 8. Unpaired two-tailed Student’s *t* test was used to analyze the differences between two groups. Comparisons among multiple groups were analyzed with one-way analysis of variance. Survival curves were compared using the log-rank test. The results are presented as means ± SD. *P* < 0.05 was considered statistically significant.

### Reporting summary

Further information on research design is available in the Nature Portfolio Reporting Summary linked to this article.

## Supplementary information

Supplementary Information

Peer Review File

Reporting Summary

## Data Availability

The sequence data generated in this study have been deposited in the GEO database under the accession codes PRJNA786718 and PRJNA786748. All the data generated in this study are provided in the Supplementary Information and Source data file. Source data are provided with this paper.

## Source data

Source Data
