# Supplementary material for: PD-L1 negatively regulates antifungal immunity by inhibiting neutrophil release from bone marrow
Source: Nat Commun. 2022 Nov 11;13:6857. doi: 10.1038/s41467-022-34722-7 (PMC9652346; doi:10.1038/s41467-022-34722-7)
Supplement: Supplementary file 1 — Supplementary Information [file 41467_2022_34722_MOESM1_ESM.pdf]

## Supplemental Figures

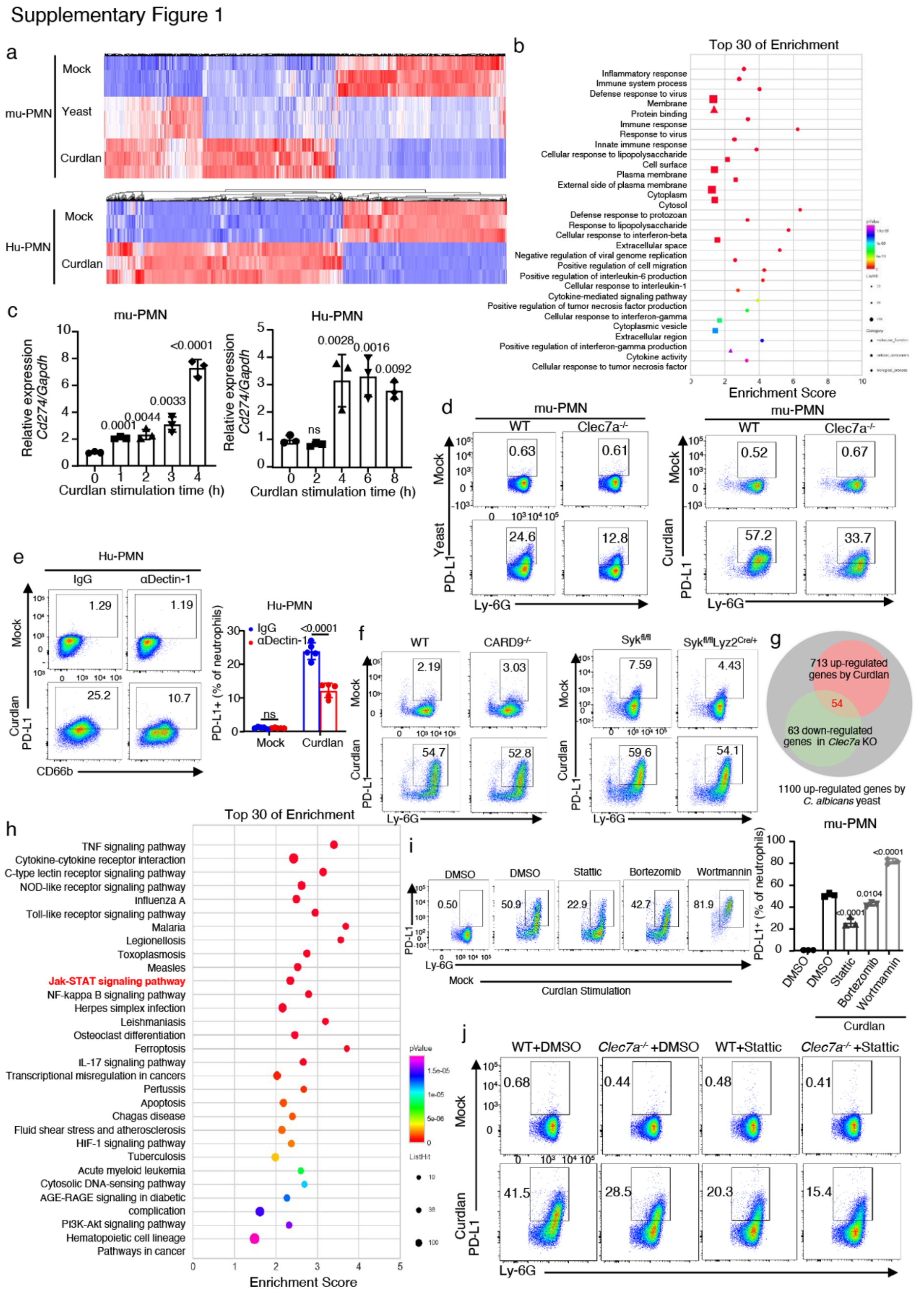

**Supplementary Figure 1. β-glucans from *C. albicans* activate the Dectin-1/JAK2/STAT3 axis to initiate PD-L1 expression in neutrophils. (a)** Heatmaps of

mu-PMNs and Hu-PMNs RNA-seq. **(b)** Gene Ontology (GO) analysis of 476 up-regulated genes in murine neutrophils and human neutrophils after stimulation with β-glucans or heat-inactivated *C. albicans* yeast as in Figure1A. **(c)** qRT-PCR analysis of PD-L1 enrichment in mu-PMNs and Hu-PMNs after stimulation with curdlan (25μg/well for mu-PMNs and 50μg/well for Hu-PMNs) for the indicated time. **(d)** Flow cytometry analysis chart of PD-L1$^{+}$ Ly-6G$^{+}$ mu-PMNs in *Clec7a*$^{-/-}$ mouse after stimulation with yeast (MOI=1) or curdlan (25μg/well) for 12 hours, **related to Fig 1e**. **(e)** The percentage of PD-L1$^{+}$ CD66b$^{+}$ Hu-PMNs treated with anti-Dectin-1 (αDectin-1, 1μg/ml), which were then stimulated with curdlan (50μg/well) for 12 hours. **(f)** Flow cytometry analysis chart of PD-L1$^{+}$ Ly-6G$^{+}$ mu-PMNs in CARD9$^{-/-}$ or Syk$^{fl/fl}$Lyz2$^{Cre/+}$ mouse after stimulation with curdlan(25μg/well) for 12 hours, **related to Fig 1f**. **(g)** Number of called peaks differentially regulated as indicated. **(h)** KEGG analysis of 54 co-upregulated genes in wild-type mu-PMNs and down-regulated in Clec7a$^{-/-}$ mu-PMNs, which were stimulated with curdlan(25μg/well) or heat-inactivated *C. albicans* yeast (MOI=0.1) for 4 hours. **(i)** The percentage of PD-L1$^{+}$ Ly-6G$^{+}$ mu-PMNs stimulated with curdlan(25μg/well) combined with inhibitors Stattic(1μM), Bortezomib(5nM) or Wortmannin(0.5μM) for 12 hours. **(j)** Flow cytometry analysis chart of PD-L1$^{+}$ Ly-6G$^{+}$ mu-PMNs in wild-type and *Clec7a*$^{-/-}$ mouse stimulated with curdlan(25μg/well) combined with inhibitor Stattic (1μM) for 12 hours, **related to Fig 1i**. Data were presented as mean ± SD, n=3(**c, i**), n=5(**e**) biological independent samples. Data were analyzed by unpaired two-sided Student's t test in **e** and one-way ANOVA adjusted for multiple comparisons in **c, i**. Source data are provided as a Source Data file.

Supplementary Figure 2

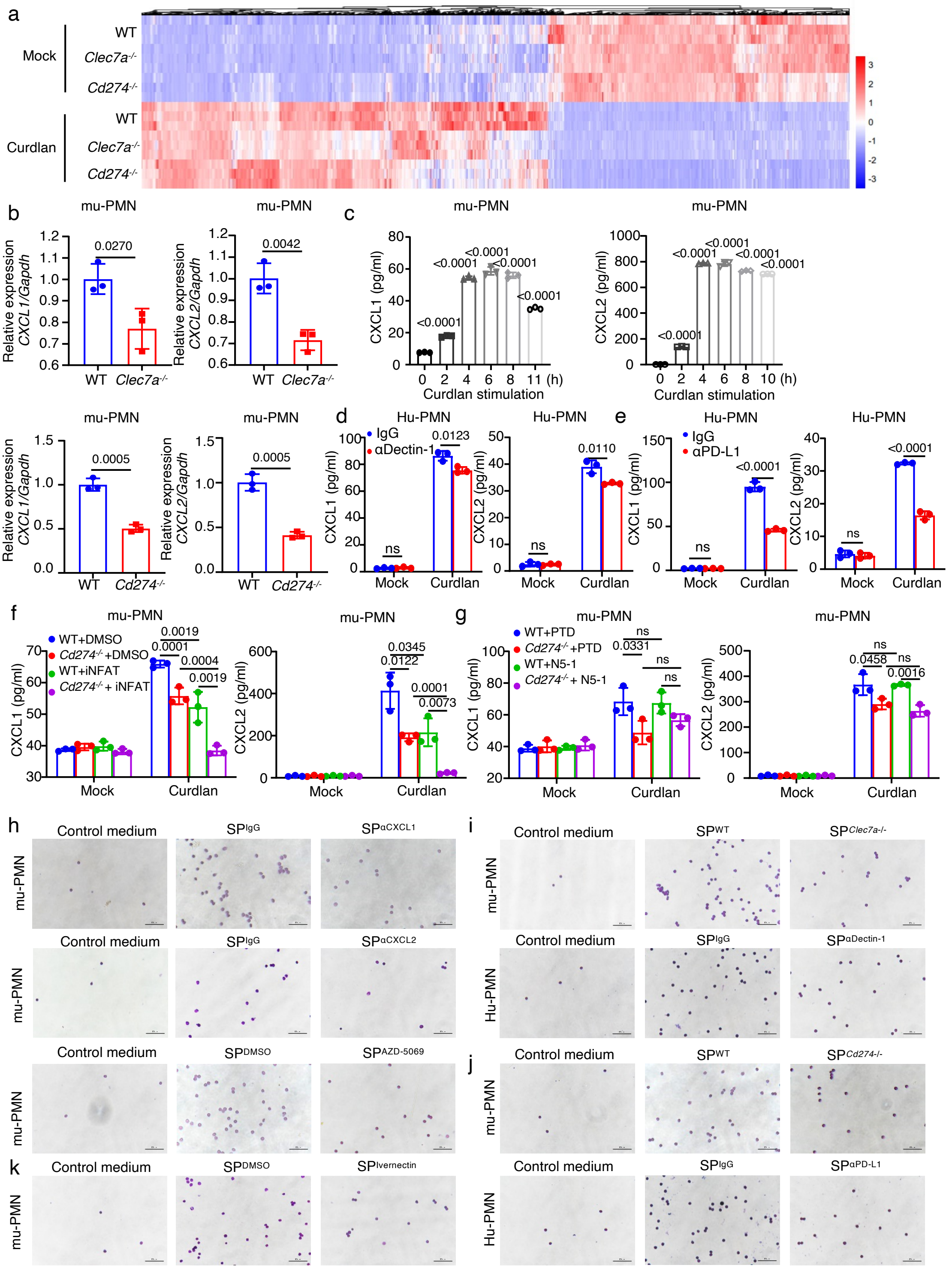


**Supplementary Figure 2. PD-L1 governs the mobilization of neutrophils through regulating their autocrine secretion of CXCL1/2. (a)** Heatmaps of mu-PMNs RNA-seq. **(b)** qRT-PCR analysis of CXCL1 and CXCL2 enrichment in *Clec7a*$^{-/-}$ and *Cd274*$^{-/-}$ mu-PMNs, which were stimulated with curdlan(25µg/well) for 4 hours. **(c)** ELISA quantification of supernatant CXCL1 and CXCL2 in mu-PMN after stimulation with

curdlan(25μg/well) for the indicated time. **(d-e)** ELISA quantification of supernatant CXCL1 and CXCL2 in Hu-PMNs after treatment with anti-Dectin-1 (αDectin-1, 1μg/ml) or anti-PD-L1 (αPD-L1, 10μg/ml), which were stimulated with curdlan (50μg/well) for 4 hours. **(f-g)** ELISA quantification of supernatant CXCL1 and CXCL2 in mu-PMN stimulated with curdlan (25μg/well) combined with inhibitor iNFAT (5μM) or N5-1 (5mM) for 4 hours. **(h-k)** Representative images of crystal violet staining of trans-well assay, **related to Fig 2l-o**. Scale bar=50 μm. Data were presented as mean ± SD, n=3(**b-g**) biological independent samples. Data were analyzed by unpaired two-sided Student's t test in **b, d-e** and one-way ANOVA adjusted for multiple comparisons in **c, f-g**. Source data are provided as a Source Data file.

Supplementary Figure 3

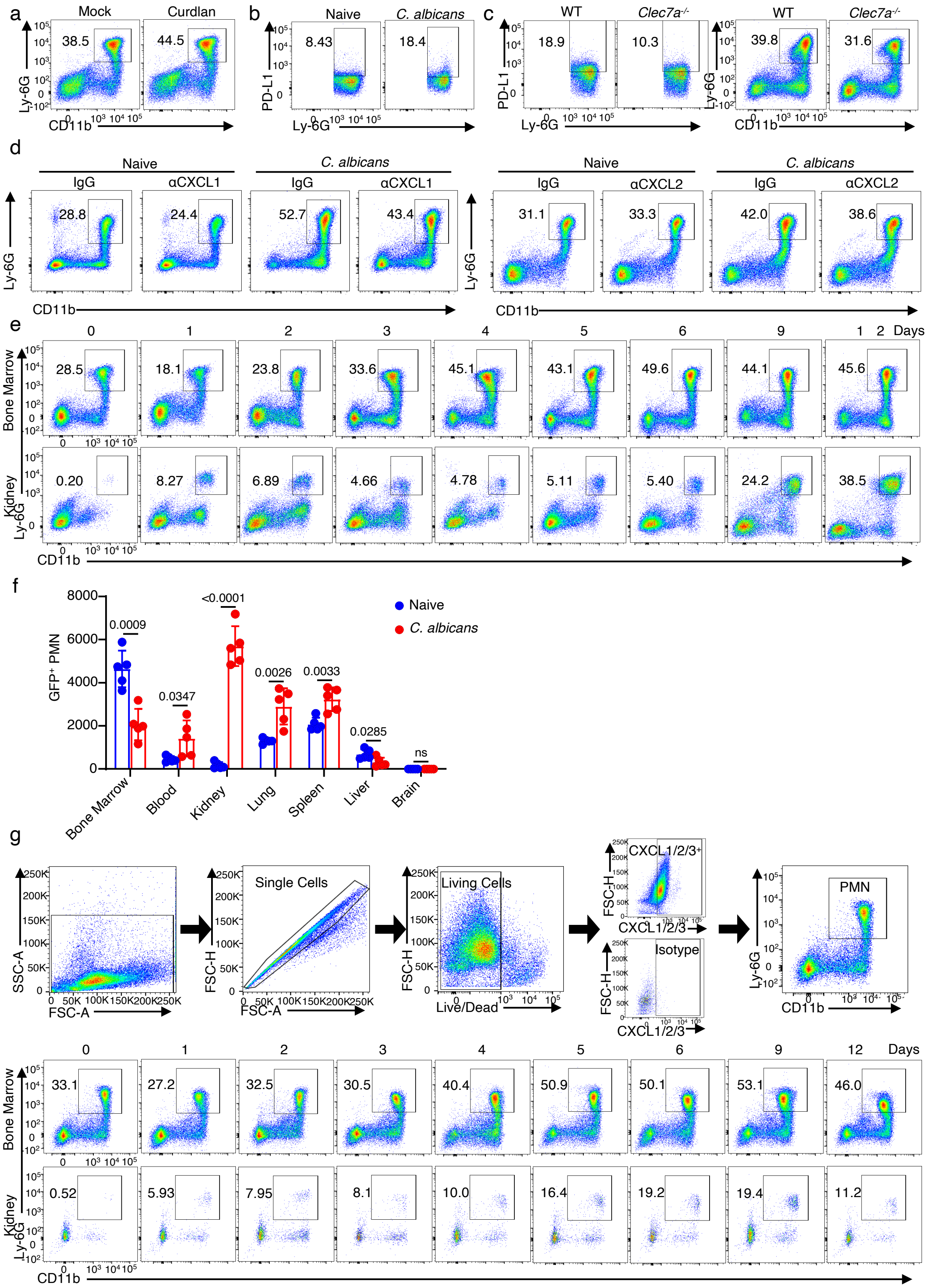


**Supplementary Figure 3. Bloodstream infection with *C. albicans* induces PD-L1 expression in neutrophils through Dectin-1 and subsequent neutrophil accumulation in the bone marrow. (a)** Flow cytometry analysis chart of neutrophils in the bone marrow of wild-type mice, which were microinjected with curdlan (0.1μg)

into the tibia for 2 days, **related to Fig 3c**. **(b)** Flow cytometry analysis chart of PD-L1$^+$Ly-6G$^+$ neutrophils in the bone marrow of wild-type mice, which were intravenously infected with 2×10$^5$ CFUs of *C. albicans* strain SC5314 for 4 days, **related to Fig 3e**. **(c)** Flow cytometry analysis chart of PD-L1$^+$Ly-6G$^+$ (Left) Ly-6G$^+$ neutrophils (Right) in the bone marrow of wild-type and *Clec7a*$^{-/-}$ mice, which were intravenously infected with 2×10$^5$ CFUs of *C. albicans* strain SC5314 for 4 days, **related to Fig 3f**. **(d)** Flow cytometry analysis chart of neutrophils in bone marrow of mock and *C. albicans* (2×10$^5$ CFU/mouse)-infected wild-type mice on day 4, which were pretreated with IgG (Control, 5 or 40ng/mouse), anti-CXCL1 (5ng/mouse) and anti-CXCL2 (40ng/mouse) into tibia for 24 hours before scarification, **related to Fig 3h**. **(e)** Flow cytometry analysis chart of neutrophils in the bone marrow and kidney of wild-type mice after intravenous infection with *C. albicans* SC5314 (2×10$^5$ CFU/mouse) for indicated days, **related to Fig 3i**. **(f)** GFP$^+$ neutrophils (2×10$^6$ cells/mouse) derived from bone marrow were intravenously infected into *C. albicans* SC5314 (2×10$^5$ CFU/mouse) infected wild-type mice for 12 hours before scarification at day 4. The absolute count of GFP$^+$ PMN in the indicated organs (one tibia, 50μl blood, one kidney, one piece of lung, half of spleen, the smallest piece of liver, one brain). **(g)** Gating strategy of Ly-6G$^+$ neutrophils among CXCL1/2/3$^+$ cells. Flow cytometry analysis chart of Ly-6G$^+$ neutrophils in CXCL1/2/3$^+$ cells in the bone marrow and kidney of *C. albicans* (2×10$^5$ CFU/mouse)-infected wild-type mice for indicated days, **related to Fig 3j**. Data were presented as mean ± SD, n=5(**f**) biological independent samples. Data were analyzed by unpaired two-sided Student's t test in **f**. Source data are provided as a Source Data file.

Supplementary Figure 4

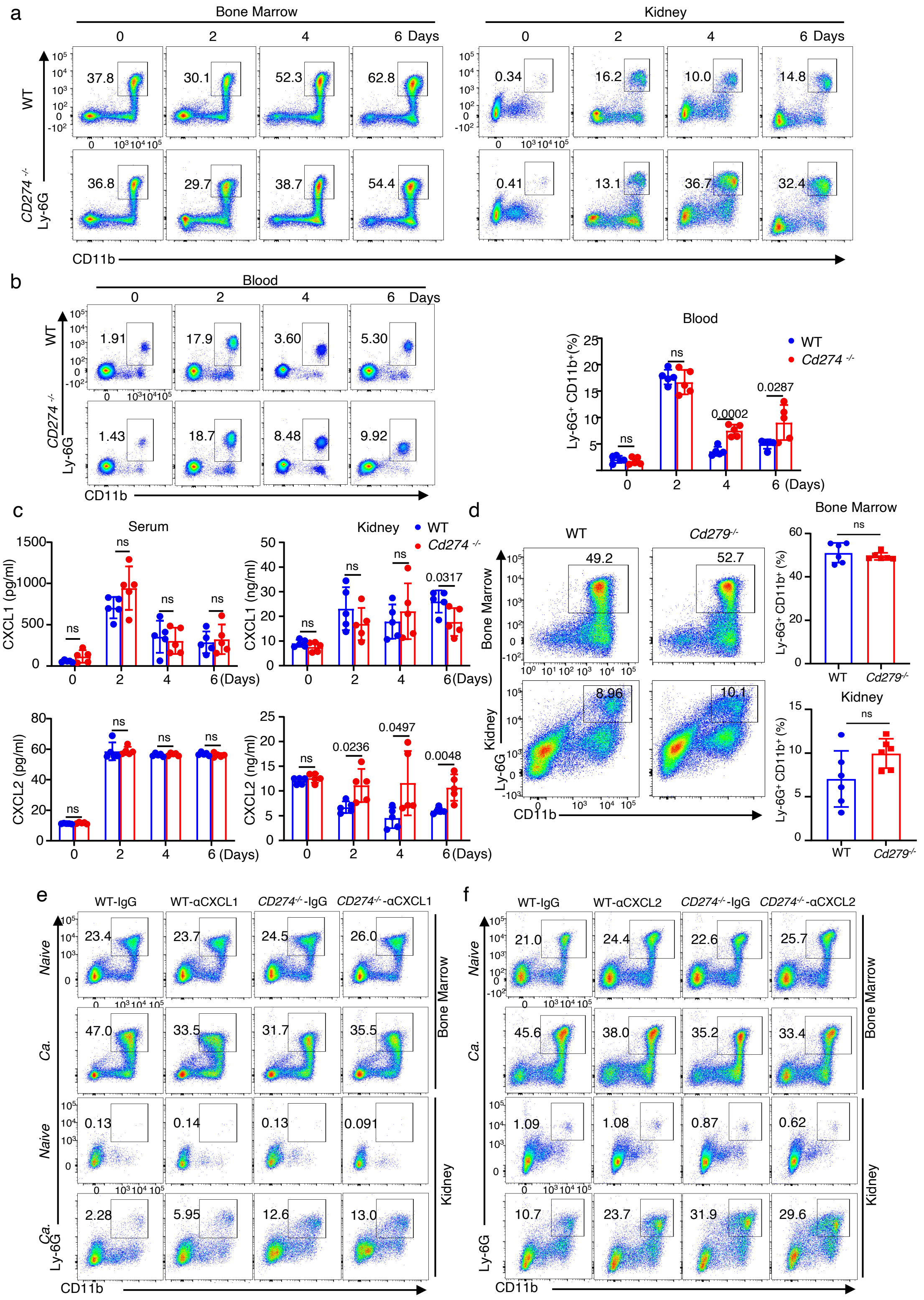


**Supplementary Figure 4. Deficiency of PD-L1 significantly increases survival of *C. albicans*-infected mice through promoting neutrophil migration from bone marrow into kidney**. **(a)** Flow cytometry analysis chart of neutrophils in the bone marrow and kidney of wild-type and *Cd274*$^{-/-}$ mice, which were intravenously infection

with *C. albicans* SC5314 ($2\times10^5$ CFU/mouse) for indicated days, **related to Fig 4b**. **(b)** The percentage of Ly-6G$^+$ neutrophils in the blood of wild-type and *Cd274*$^{-/-}$ mice, which were intravenously infection with *C. albicans* SC5314 ($2\times10^5$ CFU/mouse) for indicated days. **(c)** ELISA quantification of CXCL1 and CXCL2 in serum and kidney of wild-type and *Cd274*$^{-/-}$ mice, which were intravenously infection with *C. albicans* SC5314 ($2\times10^5$ CFU/mouse) for indicated days. **(d)** The percentage of Ly-6G$^+$ neutrophils in bone marrow and kidney of wild-type and *Cd279*$^{-/-}$ mice, which were intravenously infection with *C. albicans* SC5314 ($2\times10^5$ CFU/mouse) for 4 days. **(e-f)** Flow cytometry analysis chart of neutrophils in bone marrow and kidney of naive and *C. albicans* ($2\times10^5$ CFU/mouse)-infected wild-type and *Cd274*$^{-/-}$ mice on day 4, which were pretreated with IgG (Control, 5 or 40ng/mouse), anti-CXCL1 (5ng/mouse) and anti-CXCL2 (40ng/mouse) into tibia for 12 hours before scarification, **related to Fig 4f, h respectively**. Data were presented as mean ± SD, n=5(**b, c**), n=6(**d**) biological independent samples. Data were analyzed by unpaired two-sided Student's t test in **b-d**. Source data are provided as a Source Data file.

Supplementary Figure 5

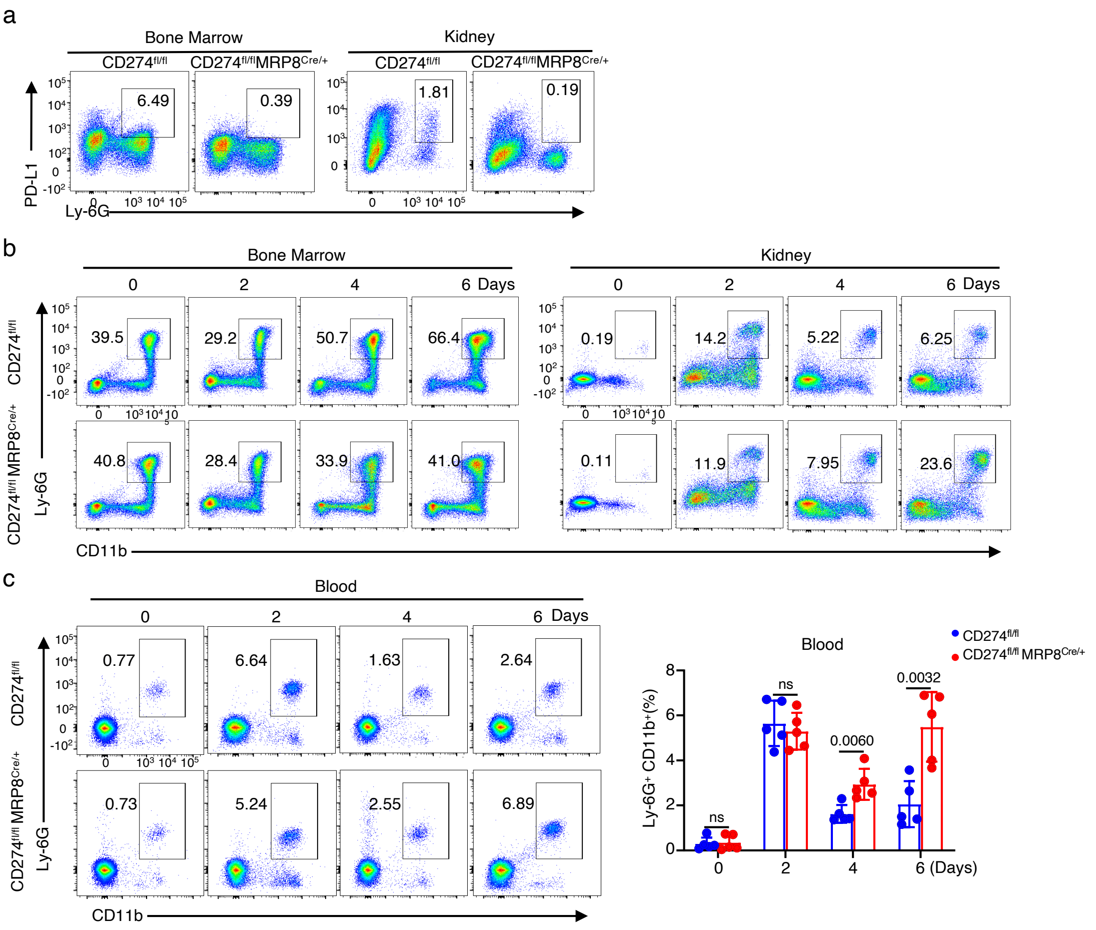


**Supplementary Figure 5. Neutrophil-specific deficiency of PD-L1 facilitates neutrophil migration from the bone marrow into the kidney of *C. albicans*-infected mice. (a)** Flow cytometry analysis chart of PD-L1$^{+}$ neutrophils in the bone marrow and kidney of CD274$^{fl/fl}$ and CD274$^{fl/fl}$MRP8$^{Cre/+}$ mice after infection with *C. albicans* SC5314 (2×10$^{5}$ CFU/mouse) on Day 4, **related to Fig 5a**. **(b)** Flow cytometry analysis chart of neutrophils in the bone marrow and kidney of CD274$^{fl/fl}$ and CD274$^{fl/fl}$MRP8$^{Cre/+}$ mice, which were intravenously infection with *C. albicans* SC5314 (2×10$^{5}$ CFU/mouse) for indicated days, **related to Fig 5c**. **(c)** The percentage of Ly-6G$^{+}$ neutrophils in the blood of CD274$^{fl/fl}$ and CD274$^{fl/fl}$MRP8$^{Cre/+}$ mice, which were intravenously infection with *C. albicans* SC5314 (2×10$^{5}$ CFU/mouse) for indicated days. Data were presented as mean ± SD, n=5(**c**) biological independent samples. Data were analyzed by unpaired two-sided Student's t test in **c**. Source data are provided as a Source Data file.

Supplementary Figure 6

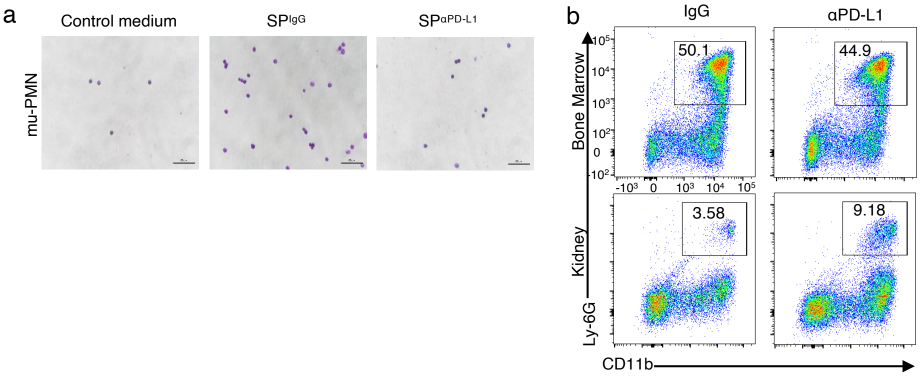


**Supplementary Figure 6. PD-L1 blockade facilitates neutrophil-based immunotherapy against lethal C. albicans sepsis. (a)** Representative images of crystal violet staining results show the trans-well data as shown in **Fig 6b** of wild-type mu-PMNs treated with anti-PD-L1 (10μg/ml), which were co-stimulated with curdlan (25μg/well) for 4 hours. **(b)** Flow cytometry analysis chart of neutrophils in the bone marrow of *C. albicans* (SC5314, $2\times10^5$ CFU/mouse)-infected wild-type mice, which were treated by IgG (Control, 200μg/mice) and anti-PD-L1 (200μg/mice) for 4 days, **related to Fig 6c**.

Supplementary Figure 7

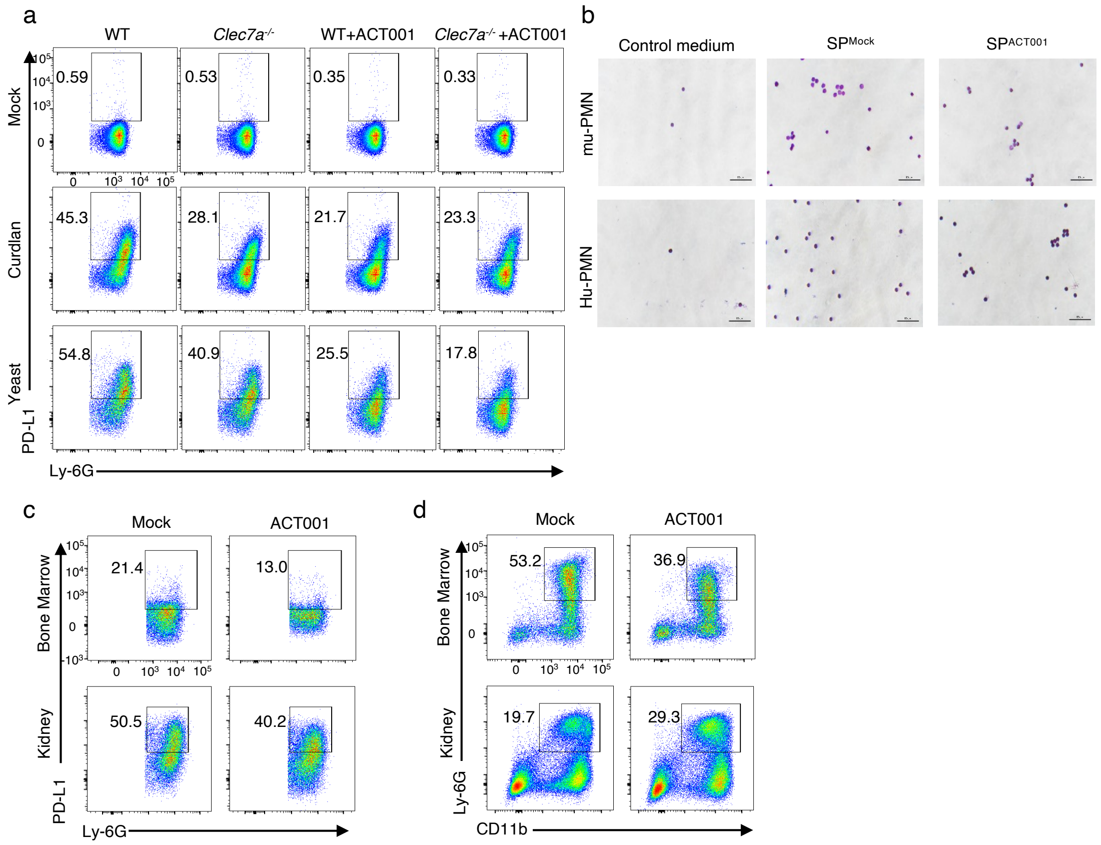


**Supplementary Figure 7. ACT001 inhibits PD-L1 expression to enhance neutrophil-mediated antifungal immunity against lethal *C. albicans* sepsis. (a)** Flow cytometry analysis chart of PD-L1$^+$ Ly-6G$^+$ mu-PMNs in wild-type and *Clec7a*$^{-/-}$ mouse stimulated with curdlan (25μg/well) or yeast (MOI=1) combined with inhibitor ACT001(40μmol/L) for 12 hours, **related to Fig 7c**. **(b)** Representative images of crystal violet staining results show the trans-well data as shown in **Fig 7f** of mu-PMNs and Hu-PMNs after treatment with ACT001 (40μmol/L), which were co-stimulated with curdlan (25μg/well for mu-PMNs and 50μg/well for Hu-PMNs) for 4 hours. **(c-d)** Flow cytometry analysis chart of PD-L1$^+$ neutrophils **(c)** and neutrophils **(d)** in the bone marrow of wild-type mice on day 4, which were intravenously infected with *C. albicans* (SC5314, 2×10$^5$ CFU/mouse)-infected wild-type mice, which were intragastrically treated with ACT001 (200 mg/kg) on day 1 and 3, **related to Fig 7g, h respectively**.

**Supplemental Tables**

**Table S1 Primer sequences of targeted genes**

| Gene | Sequences (5’-3’) |
|---|---|
| H-PD-L1 | F: 5’-TGGCATTTGCTGAACGCATTT-3’ |
| | R: 5’-TGCAGCCAGGTCTAATTGTTTT-3’ |
| M-PD-L1 | F: 5’-GCTCCAAAGGACTTGTACGTG-3’ |
| | R: 5’- TGATCTGAAGGGCAGCATTTC-3’ |
| M-CXCL1 | F: 5’-CTGGGATTCACCTCAAGAACATC-3’ |
| | R: 5’-CAGGGTCAAGGCAAGCCTC-3’ |
| M-CXCL2 | F: 5’-CCAACCACCAGGCTACAGG-3’ |
| | R: 5’-GCGTCACACTCAAGCTCTG-3’ |
| H-GAPDH | F: 5’-CTGGAGAAACCTGCCAAGTA-3’ |
| | R: 5’-TGTTGCTGTAGCCGTATTCA-3’ |
| M-GAPDH | F: 5’-CTCATGACCACAGTCCATGC-3’ |
| | R: 5’-CACATTGGGGGTAGGAACAC-3’ |
