# Supplementary material for: PD-L1 negatively regulates antifungal immunity by inhibiting neutrophil release from bone marrow
Source: Nat Commun. 2022 Nov 11;13:6857. doi: 10.1038/s41467-022-34722-7 (PMC9652346; doi:10.1038/s41467-022-34722-7)
Supplement: Supplementary file 3 — Reporting Summary [file 41467_2022_34722_MOESM3_ESM.pdf]

nature portfolio

Corresponding author(s): XIN-MING JIA

Last updated by author(s): Oct 31, 2022

Reporting Summary

Nature Portfolio wishes to improve the reproducibility of the work that we publish. This form provides structure for consistency and transparency in reporting. For further information on Nature Portfolio policies, see our Editorial Policies and the Editorial Policy Checklist.

Statistics

For all statistical analyses, confirm that the following items are present in the figure legend, table legend, main text, or Methods section.

n/a	Confirmed	
☐	☒	The exact sample size (*n*) for each experimental group/condition, given as a discrete number and unit of measurement
☐	☒	A statement on whether measurements were taken from distinct samples or whether the same sample was measured repeatedly
☐	☒	The statistical test(s) used AND whether they are one- or two-sided<br>*Only common tests should be described solely by name; describe more complex techniques in the Methods section.*
☒	☐	A description of all covariates tested
☒	☐	A description of any assumptions or corrections, such as tests of normality and adjustment for multiple comparisons
☐	☒	A full description of the statistical parameters including central tendency (e.g. means) or other basic estimates (e.g. regression coefficient) AND variation (e.g. standard deviation) or associated estimates of uncertainty (e.g. confidence intervals)
☐	☒	For null hypothesis testing, the test statistic (e.g. *F*, *t*, *r*) with confidence intervals, effect sizes, degrees of freedom and *P* value noted<br>*Give P values as exact values whenever suitable.*
☒	☐	For Bayesian analysis, information on the choice of priors and Markov chain Monte Carlo settings
☒	☐	For hierarchical and complex designs, identification of the appropriate level for tests and full reporting of outcomes
☒	☐	Estimates of effect sizes (e.g. Cohen's *d*, Pearson's *r*), indicating how they were calculated

Our web collection on statistics for biologists contains articles on many of the points above.

Software and code

Policy information about availability of computer code

Data collection	Flow cytometry data was collected by FACSCelesta (BD Biosciences) using BD DIVA8.0.1 software (BD Biosciences).<br>qPCR was done by Bio-Rad CFX96 PCR System.<br>The integrity of RNA was assessed using the Agilent 2100 Bioanalyzer (Agilent Technologies, Santa Clara). Then the libraries were constructed using TruSeq Stranded mRNA LT Sample Prep Kit (Illumina).
Data analysis	Flow cytometry data was analyzed by Flowjo v10.<br>Data analysis and plotted were using GraphPad Prism 8.

For manuscripts utilizing custom algorithms or software that are central to the research but not yet described in published literature, software must be made available to editors and reviewers. We strongly encourage code deposition in a community repository (e.g. GitHub). See the Nature Portfolio guidelines for submitting code & software for further information.

Data

Policy information about availability of data

All manuscripts must include a data availability statement. This statement should provide the following information, where applicable:

- Accession codes, unique identifiers, or web links for publicly available datasets
- A description of any restrictions on data availability
- For clinical datasets or third party data, please ensure that the statement adheres to our policy

The sequence data generated in this study have been deposited in the GEO database under the accession code PRJNA786718 (https://www.ncbi.nlm.nih.gov/bioproject/PRJNA786718) and PRJNA786748 (https://www.ncbi.nlm.nih.gov/bioproject/PRJNA786748). All the data generated in this study are provided in the Supplementary Information and Source Data file. Source data are provided with this paper.

Human research participants

Policy information about studies involving human research participants and Sex and Gender in Research.

Reporting on sex and gender	This study applied in both sexes. Sex and gender were not considered in study design.
Population characteristics	The human neutrophils were isolated from peripheral blood of 18-60 years-old healthy volunteers.
Recruitment	Participants were recruited via advertising on school web-pages and suggested by clinical doctors.
Ethics oversight	Studies of human neutrophils were approved by the Human Research Committee of Shanhai Tongji University School of Medicine (protocol No. 2021TJDX018). All participants involved in this study were informed orally and in writing of objectives, contents, risks, and discomforts associated with participation before writing informed consent.

Note that full information on the approval of the study protocol must also be provided in the manuscript.

Field-specific reporting

Please select the one below that is the best fit for your research. If you are not sure, read the appropriate sections before making your selection.

☒ Life sciences ☐ Behavioural & social sciences ☐ Ecological, evolutionary & environmental sciences

For a reference copy of the document with all sections, see nature.com/documents/nr-reporting-summary-flat.pdf

Life sciences study design

All studies must disclose on these points even when the disclosure is negative.

Sample size	The minimum of samples in each experiment was n=3, and up to n=10. The exact n for each experiment was described in corresponding figure legends. Sample sizes were determined based on expected effect size and variability within the sample, previous experience and standards in the field. Low variability between the same type of samples, indicated as SD, confirming that n=3-6 samples is sufficient to observe statistically significant differences between relevant groups. Whereas for survival rate experiments, at least n=10 samples were used as figure legend indicated.
Data exclusions	No data was excluded from analysis.
Replication	At least three independent experiments were performed for each experiments, each panel presented the representative data. All reported data were reproduced reliably.
Randomization	For animal models, all mice (WT, Clec7a-/-, Cd274-/- or Cd279-/-, Cd274 fl/fl, Cd274 fl/fl MRP8 cre/+) used in this study were divided into different treatment groups randomly. For cell culture, like Trans-well migration assay, cytokine measurement, or neutrophils for Flow Cytometry, cells were divided into each plates and assigned into different treatments groups randomly.
Blinding	All data acquisition and analysis in this study were performed in a blinded way.

Reporting for specific materials, systems and methods

We require information from authors about some types of materials, experimental systems and methods used in many studies. Here, indicate whether each material, system or method listed is relevant to your study. If you are not sure if a list item applies to your research, read the appropriate section before selecting a response.

Materials & experimental systems

n/a	Involved in the study
☐	☒ Antibodies
☒	☐ Eukaryotic cell lines
☒	☐ Palaeontology and archaeology
☐	☒ Animals and other organisms
☒	☐ Clinical data
☒	☐ Dual use research of concern

Methods

n/a	Involved in the study
☒	☐ ChIP-seq
☐	☒ Flow cytometry
☒	☐ MRI-based neuroimaging

Antibodies

Antibodies used

Antibodies used in this paper:
For Flow cytometry
PE Rat Anti-Mouse CD274 BD Biosciences 558091; 1:100
Brilliant Violet 421™ anti-mouse Ly-6G Antibody BioLegend 127628; 1:100
PerCP/Cyanine5.5 anti-mouse/human CD11b Antibody BioLegend 101228; 1:100
PE anti-mouse CD45 Antibody BioLegend 103106; 1:100
BV421 Mouse Anti-Human CD66 BD Biosciences 562741; 1:100
eBioscience™ Fixable Viability Dye eFluor™ 780 Invitrogen™ 103106; 1:100
For microscope
Anti-rabbit IgG (H+L), F(ab')2 Fragment (Alexa Fluor® 488 Conjugate) CST 4412S; 1:200
PD-L1/CD274 Monoclonal Antibody (Clone # 2B11D11) Proteintech 66248-1-Ig; 1:200
For western blot
Phospho-Jak2 (Tyr1007/1008) (C80C3) Rabbit mAb CST 3776S; 1:1000
Phospho-Stat3 (Ser727) Antibody CST 9134S; 1:1000
Jak2 (D2E12) XP® Rabbit mAb CST 3230S; 1:1000
Stat3 (D3Z2G) Rabbit mAb CST 12640S; 1:1000
Anti-PD-L1 antibody [EPR20529] Abcam ab213480; 1:1000
Rabbit anti-CD274 Polyclonal Antibody Absin abs136046; 1:1000
β-Actin (8H10D10) Mouse mAb CST 3700S; 1:1000
GAPDH (D16H11) XP® Rabbit mAb CST 5174S; 1:1000
Lamin B1 (D4Q4Z) Rabbit mAb CST 12586S; 1:1000
For Neutralization
Human Dectin-1 Neutralizing antibody - Monoclonal Mouse IgG1 (Clone # 22H8) Invivogen mabg-hdect; 1µg/ml
Mouse IgG1 isotype control (Clone # T8E5) Invivogen mabg1-ctrlm; 1µg/ml
GoInVivo™ Purified anti-mouse CD274 (B7-H1, PD-L1) Antibody (Clone # 10F.9G2) BioLegend 124328; 200µg/mice
GoInVivo™ Purified Rat IgG2b, κ Isotype Ctrl Antibody (Clone # RTK4530) BioLegend 400666; 200µg/mice
Ultra-LEAF™ Purified anti-human CD274 (B7-H1, PD-L1) Antibody (Clone # 29E.2A3) BioLegend 329715; 10µg/ml
Ultra-LEAF™ Purified Mouse IgG2b, κ Isotype Ctrl Antibody (Clone # MPC-11) BioLegend 400347; 10µg/ml
Ultra-LEAF™ Purified anti-mouse CD274 (B7-H1, PD-L1) Antibody (Clone # 10F.9G2) BioLegend 124338; 10µg/ml
Ultra-LEAF™ Purified Rat IgG2b, κ Isotype Ctrl Antibody (Clone # RTK4530) ioLegend 400671; 10µg/ml
Mouse CXCL1/GRO alpha /KC/CINC-1 Antibody (Clone # 48415) R&D Systems MAB453; 0.2µg/ml or 5ng/mice once
Rat IgG2A Isotype Control (Clone # 54447) R&D Systems MAB006; 0.2µg/ml or 5ng/mice once
Mouse CXCL2/GRO beta /MIP-2/CINC-3 Antibody (Clone # 40605) R&D Systems MAB452; 2µg/ml or 40ng/mice once
Rat IgG2B Isotype Control (Clone # 141945) R&D Systems MAB0061; 2µg/ml or 40ng/mice once

Validation

All antibodies were validated before first usage. This involve appropriate negative and positive controls.
For Flow cytometry antibodies, we relied on the manufacturer's validation which were used in many labs and published papers, and we performed isotype control when we first use, including PE Rat Anti-Mouse CD274, Brilliant Violet 421™ anti-mouse Ly-6G Antibody, APC anti-mouse/human CD11b Antibody, PE/FITC anti-mouse CD45 Antibody, BV421 Mouse Anti-Human CD66 BD.
For microscopy, detailed validation from manufacturer's data sheets:
-PD-L1/CD274 Monoclonal Antibody (Clone # 2B11D11) Proteintech 66248-1-Ig
Product is used in WB, IHC, IF, FC, ELISA applications and shows reactivity with human, mouse, pig, rat samples, the recommended concentration is 1:50-1:500
-Anti-rabbit IgG (H+L), F(ab')2 Fragment (Alexa Fluor® 488 Conjugate) CST 4412S
Product is used in published papers, the recommended concentration is 1:500 – 1:2000
For western blot, detailed validation from manufacturer's data sheets:
-Phospho-Jak2 (Tyr1007/1008) (C80C3) Rabbit mAb CST 3776S
Western blot analysis of extracts from UT-7 and BaF3 cells, untreated or treated with GM-CSF or IL-3 (5 minutes), using Phospho-Jak2 (Tyr1007/1008) (C80C3) Rabbit mAb #3776 (top) or total Jak2 (D2E12) XP® Rabbit mAb #3230 (bottom).
-Phospho-Stat3 (Ser727) Antibody CST 9134S
Western blot analysis of extracts from A172 cells, untreated (-) or UV-treated (100 mJ, 30 min; +) with or without λ phosphatase (+), using Phospho-Stat3 (Ser727) Antibody (upper) and Stat3 (79D7) Rabbit mAb #4904 (lower).
-Jak2 (D2E12) XP® Rabbit mAb CST 3230S
Western blot analysis of extracts from K-562 cells, transfected with 100 nM SignalSilence® Control siRNA (Unconjugated) #6568 (-) or Jak2 siRNA (+).
-Stat3 (D3Z2G) Rabbit mAb CST 12640S
Western blot analysis of extracts from various cell lines using Stat3 (D3Z2G) Rabbit mAb (upper), or β-Actin (D6A8) Rabbit mAb #8457 (lower). PC-3 cells are negative for Stat3.
-Anti-PD-L1 antibody [EPR20529] Abcam ab213480

Untreated RAW264.7 (mouse Abelson murine leukemia virus-induced tumor macrophage) whole cell lysate.
-Rabbit anti-CD274 Polyclonal Antibody Absin abs136046
CD274 Antibody detects endogenous levels of total CD274.
For Neutralization
Anti-hDectin-1-IgG is a monoclonal mouse IgG1 antibody against human Dectin-1 (hDectin-1). This antibody was screened for neutralization activity and flow cytometry. This antibody was widely used in many labs and validated by published papers.
GoInVivo™ Purified anti-mouse CD274 (B7-H1, PD-L1) Antibody and Ultra-LEAF™ Purified anti-human CD274 (B7-H1, PD-L1) Antibody were Neutralization of a soluble factor by an antibody. These antibodies were widely used in many labs and validated by published papers. We also validated their neutralizing function by using isotype.
These neutralizing antibodies, Human Dectin-1 Neutralizing antibody - Monoclonal Mouse IgG1 (Clone # 22H8), GoInVivo™ Purified anti-mouse CD274 (B7-H1, PD-L1) Antibody (Clone # 10F.9G2), Ultra-LEAF™ Purified anti-human CD274 (B7-H1, PD-L1) Antibody (Clone # 29E.2A3), Ultra-LEAF™ Purified anti-mouse CD274 (B7-H1, PD-L1) Antibody (Clone # 10F.9G2), Mouse CXCL1/GRO alpha /KC/ CINC-1 Antibody (Clone # 48415) and Mouse CXCL2/GRO beta /MIP-2/CINC-3 Antibody (Clone # 40605), were validated by published papers. We also validated their neutralizing function by using isotype Mouse IgG1 isotype control (Clone # T8E5) Invivogen mabg1-ctrlm, GoInVivo™ Purified Rat IgG2b, κ Isotype Ctrl Antibody (Clone # RTK4530), Ultra-LEAF™ Purified Mouse IgG2b, κ Isotype Ctrl Antibody (Clone # MPC-11), Ultra-LEAF™ Purified Rat IgG2b, κ Isotype Ctrl Antibody (Clone # RTK4530), Rat IgG2A Isotype Control (Clone # 54447) and Rat IgG2B Isotype Control (Clone # 141945).

Animals and other research organisms

Policy information about studies involving animals; ARRIVE guidelines recommended for reporting animal research, and Sex and Gender in Research

Laboratory animals	C57BL/6 mice were purchased from Gempharmatech Co., Ltd (Nanjing, China). Dectin-1 and CARD9 knockout (KO) mice were from X. Lin (Tsinghua University School of Medicine, Beijing, China). Cd274 KO mice were from L. Zhou (NHFPC Key Laboratory of Combined Multi-organ Transplantation, Hangzhou, China). Cd279 KO mice were from Y. Yu (Tongji University, Shanghai, China). Sykfl/flLyz2Cre/+ mice were from H. Xiao (Institute Pasteur of Shanghai, China). CD274fl/fl and MRP8Cre/+ mice were purchased from GemPharmatech. All animal studies were performed with sex- (female and male) and age-matched (6-8weeks) mice. All mice were bred under specific pathogen-free conditions in ventilated cages with ad libitum food and water at Tongji University School of Medicine (Shanghai, China).Housing conditions were as following: dark/light cycle 12/12, ambient temperature around 21-22°C and humidity between 40 and 70%.
Wild animals	No wild animals were involved in this study.
Reporting on sex	This study applied in both sexes and sex was not considered in study design.
Field-collected samples	This study did not involve field-collected samples.
Ethics oversight	All animal studies were performed with the approval from the Institutional Laboratory Animal Care and Use Committee of Tongji University School of Medicine (Shanghai, China) (protocol No. TJAA09021102).

Note that full information on the approval of the study protocol must also be provided in the manuscript.

Flow Cytometry

Plots

Confirm that:

☒ The axis labels state the marker and fluorochrome used (e.g. CD4-FITC).

☒ The axis scales are clearly visible. Include numbers along axes only for bottom left plot of group (a 'group' is an analysis of identical markers).

☒ All plots are contour plots with outliers or pseudocolor plots.

☒ A numerical value for number of cells or percentage (with statistics) is provided.

Methodology

Sample preparation	Single-cell suspensions from kidneys were obtained by collagenase digestion. For enriching immunocytes in kidney, cells were centrifuged by using 40% and 80% Percoll gradients and harvested cell layer between them. Cells were stained with fluorochrome-conjugated antibodies, according to the manufacturer's protocols. Flow cytometry was performed with a BD FACSFertasa flow cytometry system (BD Biosciences, San Jose, Calif), and data were analyzed with FlowJo software (Tree Star, Ashland, Ore).
Instrument	FACSCelesta (BD Biosciences)
Software	Flow cytometry data was analyzed by Flowjo v10.<br>Data analysis and plotted were using GraphPad Prism 8.<br>BD DIVA8.0.1 software (BD Biosciences)
Cell population abundance	The abundance is depended on the specific population,from 0-80%.

Gating strategy	All the gating strategy for each experiment was supplied in supplementary figures. They were determined by single color stains where applicable. Generally, the first gating (FSC/SSC) was done on alive cells to exclude debris and dead cells, followed by the section of single cells (FSC-W/FSC-H), excluding clumps. Then the specific fluorophores were confirmed and analyzed by comparing single color stains.

☒ Tick this box to confirm that a figure exemplifying the gating strategy is provided in the Supplementary Information.
