# Supplementary material for: PD-L1 negatively regulates antifungal immunity by inhibiting neutrophil release from bone marrow
Source: Nat Commun. 2022 Nov 11;13:6857. doi: 10.1038/s41467-022-34722-7 (PMC9652346; doi:10.1038/s41467-022-34722-7)
Supplement: Supplementary file 2 — Peer Review File [file 41467_2022_34722_MOESM2_ESM.pdf]

nature portfolio

Peer Review File

PD-L1 negatively regulates antifungal immunity by inhibiting neutrophil release from bone marrow

Open Access This file is licensed under a Creative Commons Attribution 4.0 International License, which permits use, sharing, adaptation, distribution and reproduction in any medium or format, as long as you give appropriate credit to the original author(s) and the source, provide a link to the Creative Commons license, and indicate if changes were made. In the cases where the authors are anonymous, such as is the case for the reports of anonymous peer reviewers, author attribution should be to 'Anonymous Referee' followed by a clear attribution to the source work. The images or other third party material in this file are included in the article's Creative Commons license, unless indicated otherwise in a credit line to the material. If material is not included in the article's Creative Commons license and your intended use is not permitted by statutory regulation or exceeds the permitted use, you will need to obtain permission directly from the copyright holder. To view a copy of this license, visit http://creativecommons.org/licenses/by/4.0/.

Editorial Note: Parts of this Peer Review File have been redacted as indicated to remove third-party material where no permission to publish could be obtained.

REVIEWER COMMENTS

Reviewer #1 (Remarks to the Author):

In this manuscript, the authors delineate a novel pathway by which neutrophils are activated via fungal beta-glucans binding to Dectin-1, which drives a JAK2/STAT3 pathway to upregulate expression of PD-L1. They show that PDL1 translocates to the nucleus to mediate expression of chemokines CXCL1/CXCL2, which in turn cause neutrophils to accumulate within the bone marrow via autocrine signalling. By blocking this pathway (which the authors do using knock-out mice and chemical inhibitors), mice had better survival when challenged with systemic Candida infection, which the authors attributed to better neutrophil efflux from bone marrow and recruitment into the kidney, leading to a reduction in fungal infection in this tissue. Overall, I mostly liked this paper and think it is well presented with good controls and some convincing data. It is on an important topic and the authors use neutrophil-specific knock-out mice as well as clinically relevant drugs in their preclinical animal models to demonstrate that PDL1 expression by PMN is relevant and important for antifungal immunity. I think it will be well received by the field. My main issue with the current manuscript is that some of the in vivo data is at odds with published data from the field and this is not really explained or discussed in the current manuscript (i.e. important citations are missing). Specific comments:

1. In Fig 3I, the authors show that PMN levels fall in the kidney over time after Candida infection, while increasing in the bone marrow. However, many other studies show that neutrophils accumulate in the kidney over time and while they are indeed required to control infection, they also drive pathology in this tissue at later time points due to their massive recruitment (see Lionakis et al JII 2011 and Lionakis et al Plos Pathogens 2012). The authors don't discuss this earlier work or explain why neutrophils are falling in their model. I'm therefore unconvinced by their mechanistic explanation because of this.
2. Related to above, if the PDL1 KO mice have increased PMN recruitment to the kidney and less fungal CFU (which makes sense), I'm curious as to why the authors think the PDL1 KO kidney has more neutrophils to start with if there is no difference in the chemokines? The explanation provided is that you have better efflux from the bone marrow as there are less PMN in the marrow in the KO mice. However, they don't show blood PMN counts (which would give further evidence of PMN efflux from the marrow), and they show that there is no difference in the main PMN chemoattractants in the PDL1 KO kidney (CXCL1/2). If there is no difference in chemokines, then what is mediating their recruitment? Just because there is more efflux from marrow, it doesn't then make sense that the PMN would just go to kidney… the infection will be taking hold in liver/spleen/brain as well. Is there another chemokine rising in the PDL1 KO kidney that is driving the increased PMN recruitment? Or increased blood flow with increased circulating PMN? Some more work and discussion is needed around these points in my opinion.
3. Ageing PMN have been shown to express PDL1. Do the authors think their PDL1+ PMN are an aged population that accumulate during fungal infection? Can they look for aging signatures in their RNAseq dataset or stain for other markers of aged PMN (e.g. CXCR4)? Aged PMN have quite different functional properties so it would be interesting to see if this poorly understood population are what is being detected in the bone marrow of infected mice.

Minor comments:

1. Fig 2J; the PE labelling of PMN in the kidney is not very convincing. How are the gates being set? Can the authors please provide a negative control so that positive staining can be proven?
2. The western blots and FACS for JAK2/STAT3 are shown as representative data – but this is not stated. Can the authors please provide quantitative data or state how many times this experiment was done (i.e. how reproducible is the result)?
3. RNA seq data needs deposited.

Reviewer #2 (Remarks to the Author):

In this robust paper, Yao Yu et al show that during C. albicans infection, PD-L1 governs the mobilization of neutrophils through regulating their autocrine secretion of CXCL1 and CXCL2. In support of the above mechanism, they present that deficiency or blockade of PD-L1 significantly

impairs the secretion of CXCL1 and CXCL2 in murine or human neutrophils induced by â-glucans, and subsequently suppresses neutrophil mobilization as a consequence. This is a nice, multilayered, and novel story, that extends to the "hot" topic of the role of Check point blockage and fungal immunity. Although the upregulation of the PD-1/PD-L1 pathway and protective effects of PD-1 pathway inhibitors have been previously shown in murine models of invasive candidiasis/Candida sepsis, this study provides a first deep-dive mechanistic exploration of the involvement of PD-L1 in immune cell recruitment to the site of infection. Part of the potential significance of this study is its focus on the autocrine downstream effects of PD-L1. Most cancer immunology studies rather focused on the upstream regulation of PD-L1 expression and the implications of PD-L1 expression for interactions with other immune cells (especially lymphocytes), whereas PD-L1 effects in neutrophils are relatively scarcely studied. However, many unanswered questions remain regarding both the signaling cascades resulting in beta-glucan-induced PD-L1 upregulation and the specific downstream effects. It also remains unclear how PD-L1 specifically retains neutrophils in the bone marrow, as the in vitro experiments showed an effect of PD-L1 deficiency on CXCL1/2 production in both (murine) bone marrow derived neutrophils and (human) peripheral blood neutrophils. Furthermore, some critical links between the findings have not been sufficiently proven experimentally. Most significantly, in order to demonstrate that the therapeutic effect of PD-L1 against invasive candidiasis predominantly relies on the CXCL1/2-dependent retention of neutrophils in the bone marrow, the authors should use a CXCL1/2-deficient mouse strain and show that the therapeutic effect of PD-L1 significantly vanishes compared to CXCL1/2-producing mice.. Finally, he biggest issue with the paper is the " timing question as assessments were mostly static, whereas these are highly dynamic processes. Studying these aspects serially and get a better feeling of the kinetics to make inferences regarding the optimal timing of combinatorial strategies. Consider discussing those issues as a limitation

Specific comments:
• As indicated above, it remains entirely unclear from the authors' data why the PD-L1 effect on neutrophil attraction/retention is specifically seen in the bone marrow. In fact, the authors show PD-L1-dependent Curdlan-induced CXCL1/2 secretion in vitro in neutrophils obtained from peripheral human blood. Therefore, the question arises why PD-L1 only mediates the CXCL1/2-dependent retention of neutrophils in the bone marrow as opposed to, for example, active CXCL1/2-dependent recruitment of neutrophils to the kidney or into the bloodstream? This question is neither experimentally addressed nor sufficiently discussed. Of note studies have shown that the pharmacological Blockade of the Chemokine Receptor CCR1 Protects Mice from Systemic Candidiasis (see Antimicrob Agents Chemother . 2017 Feb 23;61(3):e02365-16. doi: 10.1128/AAC.02365-16)
• Lines 131-132 and Fig. 1B: Were other checkpoint/exhaustion markers upregulated as well or did CD274/PD-L1 stand out as the only/strongest checkpoint signal?
• Many effects shown in Fig. 1 and 2 are based on a static assessment of a single timepoint. Ideally, a more dynamic assessment to show that PD-L1 upregulation on the cell surface of neutrophils precedes the downstream effects on migration and CXCL1/2 expression would make the story line stronger.
• Multiple distinct downstream signaling pathways of Dectin-1 have been described and it remains unclear from the data presented how exactly Dectin-1 signaling leads to PD-L1 induction via JAK2 and STAT3 phosphorylation. The described Dectin-1 signaling pathways either rely on Syk, NF-kB, and/or PI3K (see a recent detailed review, PMID: 35237264). The authors show data (knockout mice or inhibitors) suggesting that all 3 proteins are not essential for PD-L1 induction. Therefore, PD-L1 induction either relies on multiple, redundant pathways, or a previously unknown pathway, but this is overall quite unclear from the authors' data (and not further discussed). It is also possible that indirect effects, e.g., through an interferon signaling loop, might be involved.
• Fig. 2H: This experiment does not necessarily point to an involvement of PD-L1. Ivermectin blocks nuclear import in an unspecific manner and would also block other potentially critical nuclear factors involved in Dectin-1 signaling, e.g., IRF5 or NFAT.
• Does PD-L1 expression affect other (direct) neutrophil effector functions that are relevant for fungal clearance, e.g., oxidative burst or NET formation?
• The focus on only two compartments (kidney and bone marrow) is an over-simplification of the complex regulation of neutrophil recruitment. For instance, others have previously shown that PD-L1-expressing neutrophils can transiently arrest in the lung vasculature, possibly contributing to injury-induced infection susceptibility (PMID: 33674305). The spleen might be another highly

pertinent repertoire. Therefore, more dynamic studies and assessment of the accumulation of labelled neutrophils in additional organs will be needed to better understand the complex dynamics. Consider discussing as a limitation

• The conclusions in lines 255-257 and 303-304 do not fully align with the raw data. Fig. 3I-J suggest that neutrophils are initially not retained in the bone marrow but recruited to the infected tissue despite PD-L1 expression. From the data presented, it rather seems that PD-L1 might impact the secondary re-distribution between peripheral tissues and bone marrow (between days 2 and 4). This would be more difficult to align with the in vitro studies presented. Unfortunately, dynamic studies with multiple time points are lacking in the experiments with PD-L1 deficient mice and the PD-L1 inhibitor (Fig. 4 and 5). Therefore, it is difficult to fully appreciate the role of PD-L1 in the initial and secondary neutrophil distribution kinetics.

• From the data presented, it ultimately remains unclear that the therapeutic effect of PD-L1 against invasive candidiasis predominantly relies on CXCL1/2-dependent neutrophil migration. It is noteworthy that the mortality advantage of anti-PD-L1 is only seen after 5-10 days, whereas the effects on neutrophil migration are seen earlier. Therefore, it remains unclear whether the therapeutic benefit relies on a) a delayed protective effect of increased neutrophil accumulation in the kidneys and/or b) additional independent mechanisms such as improved lymphocyte exhaustion. The authors could potentially add other (earlier) surrogates of protection, e.g. morbidity scores after 2 and 4 days or weight curves. These surrogates of protection usually precede the survival benefit. Furthermore, to further support a direct role of CXCL1/2 in anti-PD-L1-mediated protection, the authors should use a CXCL1/2-deficient mouse strain and show that the therapeutic effect of PD-L1 significantly vanishes compared to CXCL1/2-producing mice.

General comments

• The in vivo studies are mostly based on small numbers of mice, performed with a single Candida isolate. This should be acknowledged as a limitation.

• Several of the figure legends state that "representative dot plots from 3 independent experiments" are shown. Does this mean that multiple replicate experiments were performed but only one is shown? If so, this should be changed by showing all data in order to provide a sense of reproducibility of the results in independent experiments with cells from different donors/mice.

• Avoid the overuse of "dramatic" to describe differences between experimental cohorts/conditions. Instead, the authors could use "significant" (if applicable) or refer to the actual effect size (e.g., a 10-fold increase in…).

• Although prior studies on PD-1/PD-L1 pathway blockade in fungal infection models have been cited in the Introduction, the authors should refer to these studies in the Discussion and put their results into a broader context, e.g., by discussing additional mechanisms likely involved in checkpoint inhibitor-mediated protection against fungal infections (e.g., reduced lymphocyte exhaustion and improved maturation and fungicidal activity of mononuclear phagocytes).

• Some of the panels are blurry (e.g., Fig. 1G, 2J).

Reviewer #3 (Remarks to the Author):

The authors report that PD-L1 regulate anti-fungal response by regulating the neutrophil migration.

Other recent reports have demonstrated that PD-L1 can be induced in innate cells like dendritic cells upon stimulation with glucans or fungi like Aspergillus fumigatus. Also, PD-L1 affects the T cell responses as well (PMID: 34781737, 28968869). Unfortunate that authors have not given due credit to those reports. PD-L1 thus can regulate the anti-fungal immunity by several mechanisms and not just by regulating the neutrophil migration.

Fig 1:

CLEC7A deficient neutrophils though showed reduced PD-L1 upon stimulation with curdlan; PD-L1 expression is still high. Also, the human data of using anti-Dectin-1 blocking antibodies is not convincing

Needed to validate the results on PD-L1 expression with live candida

The lack of involvement of Syk or CARD9 need further probing on the pathway implicated. Otherwise, the data are not complete
Fig 1J: pJAK2 spectra are not convincing
Fig 1G: Give the precise pathway implicated by data mining of the RNA seq data instead of listing which are upregulated

Fig 2:
The effect of PD-L1 deficiency on CXCL1 secretion of murine neutrophils and conclusions made are not convincing
Why PD-L1 deficiency should lead to downregulation of genes including CXCL1 and CXCL2 in neutrophils upon stimulation with b-glucan? It is not a receptor for b-glucan. There are no ligands present in the experiments which signal through PD-L1. Showing that PD-L1 translocate into the nucleus does not prove that it affects the production of CXCL1 and CXCL2 in neutrophils. Need to show whether PD-L1 binds the promoter region of CXCL1 and CXCL2 genes.
What is the effect of PD-L1 or Dectin-1 deficiency on the expression of CXCR2 on the neutrophils?

Fig 3: Dectin-1 and PD-L1 are expressed on many innate cells and the use of KO mice not specific for the neutrophils does not validate the results. The effects are also possibly due to the other immune cells rather than the direct effect on the neutrophils. What is the repercussion of migration of other innate cells like monocytes, macrophages, dendritic cells to the bone marrow in all these different conditions and experiments ? Also CXCL1/2 are produced by other cells as well.

Fig 4A-D: What is the mechanism and pathway by which PD-L1 promotes neutrophil migration to the kidneys from the bone marrow ? Of note at Day 2, no influence of PD-L1 for the neutrophil migration to the kidney and effect is visible on day 4 ? Why ?

Fig 4J: what is the effect of neutrophil specific KO of PD-L1 on the survival of mice ?

Fig 5: The effect of blockade of other innate cells PD-L1 cannot be ruled out

Fig 6A: pJAK2 data are not convincing

We would like to thank editor and reviewers for their critical and constructive comments and suggestions. Based on their comments, we have performed all of the requested experiments and revised our manuscript. The following is the point-to-point responses to their comments.

Reviewer #1 (Remarks to the Author):

In this manuscript, the authors delineate a novel pathway by which neutrophils are activated via fungal beta-glucans binding to Dectin-1, which drives a JAK2/STAT3 pathway to upregulate expression of PD-L1. They show that PDL1 translocates to the nucleus to mediate expression of chemokines CXCL1/CXCL2, which in turn cause neutrophils to accumulate within the bone marrow via autocrine signalling. By blocking this pathway (which the authors do using knock-out mice and chemical inhibitors), mice had better survival when challenged with systemic Candida infection, which the authors attributed to better neutrophil efflux from bone marrow and recruitment into the kidney, leading to a reduction in fungal infection in this tissue. Overall, I mostly liked this paper and think it is well presented with good controls and some convincing data. It is on an important topic and the authors use neutrophil-specific knock-out mice as well as clinically relevant drugs in their preclinical animal models to demonstrate that PDL1 expression by PMN is relevant and important for antifungal immunity. I think it will be well received by the field. My main issue with the current manuscript is that some of the in vivo data is at odds with published data from the field and this is not really explained or discussed in the current manuscript (i.e. important citations are missing).

Specific comments:

1. In Fig 3I, the authors show that PMN levels fall in the kidney over time after Candida infection, while increasing in the bone marrow. However, many other studies show that neutrophils accumulate in the kidney over time and while they are indeed required to control infection, they also drive pathology in this tissue at later time points due to their massive recruitment (see Lionakis et al JII 2011 and Lionakis et al Plos Pathogens 2012). The authors don't discuss this earlier work or

explain why neutrophils are falling in their model. I'm therefore unconvinced by their mechanistic explanation because of this.

Response: We appreciate the reviewer for bringing these highly relevant references to our attention. Regarding your concern that neutrophil levels fall in the kidney over time after *Candida* infection in our model, we repeated these data with more testing time (2×10^5 CFU/mouse) (Fig.R1A) and these data were found to be reproducible. We further compared our data with those reported in previous studies and found that dynamic changes of neutrophil levels in the kidneys of *C. albicans* (2×10^5 CFU/mouse)-infected mice were curvilinear over time. From Day 1 to Day 2, the absolute number of neutrophils reached the first peak (~5×10^5/kidney and ~1×10^6/kidney, respectively, Fig.R1A right panel), which was consistent with the data reported by Nur et al. (2×10^5 CFU/mouse, Fig.R1B, left lane in each panel cited from *PLoS Pathog, 2019, 15, e1008115*). And then the absolute number of neutrophils fell on Day 3 and sustained with minor fluctuation from Day 3 to Day 6 (~1×10^5/kidney, Fig.R1A right panel), which was consistent with the data reported by Guiducci et al. (1.5×10^5 CFU/mouse, Fig.R1C, left lane in each panel cited from *Front Immunol, 2018, 9, 1573*). A higher dose (2.5×10^5 CFU/mouse) was used to construct infectious model by Lionakis et al. , showing that the absolute number of neutrophils increased from ~3×10^5/kidney on Day 3 to ~9×10^5/kidney on Day 6 (Fig.R1D black line in each panel cited from *PLoS Pathog, 2012, 8, e1002865*). They also showed that the absolute number of neutrophils increased from ~2×10^5/kidney on Day 1 to ~1×10^6/kidney on Day 4 (Fig.R1E cited from *J Innate Immun, 2011, 3, 180-199*). Therefore, we believe that infectious doses of *C. albicans* have great influences on the accumulation of neutrophils in kidney.

Importantly, to determine whether bloodstream infection with *C. albicans* could affect neutrophil migration from the bone marrow into kidney, we applied the cell tracking approach and found that, on Day 2 after *C. albicans* infection, the frequency and number of PE-labelled neutrophils derived from the bone marrow were significantly increased in the kidneys of mice (Fig. 3k). However, on Day 4

and 6 after *C. albicans* infection, the frequency and number of PE-labelled neutrophils in the kidney were dramatically decreased compared with those on Day 2 (Fig. 3k). Thus, these data implied that *C. albicans* infection induced neutrophil accumulation in the bone marrow through inhibiting neutrophil migration into the circulation.

A

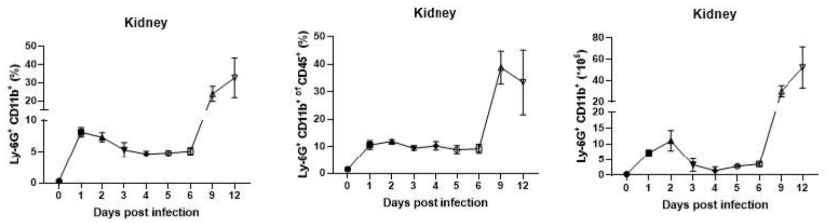

[REDACTED]

Fig R1 (A) The percentage and numbers of neutrophils, in the kidneys of wild-type mice, which were intravenously infected with 2×10^5 CFUs of *C. albicans* strain SC5314 for 12 days (n=6 for each group). **(B)** The absolute numbers of neutrophils per kidney at 24h and 48h post infection, cited from *PLoS Pathog, 2019, 15, e1008115, SFig1-A, Fig5B.* **(C)** $CD45^+$ $Ly6C^{int}$ $Ly6G^+$ neutrophils were quantified in the kidney by flow cytometry on day 3 and day 7 post-infection, cited from *Front Immunol, 2018, 9, 1573, Fig5B.* **(D)** The numbers and percent of $Ly6c^{int}Ly6G^+CD11b^+$ neutrophils in total $CD45^+$ leukocytes in $Ccr1^{+/+}$ and $Ccr1^{-/-}$ kidneys at different time-points after Candida infection. cited from *PLoS Pathog, 2019, 15, e1008115, Fig4C-D.* **(E)** Neutrophils as percent of $CD45^+$ cells, and as absolute numbers per kidney, cited from *J Innate Immun, 2011, 3, 180-199, Fig3C.*

2. Related to above, if the PDL1 KO mice have increased PMN recruitment to the kidney and less fungal CFU (which makes sense), I'm curious as to why the authors think the PDL1 KO kidney has more neutrophils to start with if there is no

difference in the chemokines? The explanation provided is that you have better efflux from the bone marrow as there are less PMN in the marrow in the KO mice. However, they don't show blood PMN counts (which would give further evidence of PMN efflux from the marrow), and they show that there is no difference in the main PMN chemoattractants in the PDL1 KO kidney (CXCL1/2). If there is no difference in chemokines, then what is mediating their recruitment? Just because there is more efflux from marrow, it doesn't then make sense that the PMN would just go to kidney… the infection will be taking hold in liver/spleen/brain as well. Is there another chemokine rising in the PDL1 KO kidney that is driving the increased PMN recruitment? Or increased blood flow with increased circulating PMN? Some more work and discussion is needed around these points in my opinion.

Response: Thank you for your good suggestions. Regarding to your concern that PD-L1 KO kidney has more neutrophils after *C. albicans* infections, we explored the tissue distribution of neutrophils after releasing from bone marrow. First, we adoptively transferred GFP$^+$ neutrophils into *C. albicans*-infected mice, and found that kidney was the organ most infiltrated by GFP$^+$ neutrophils, rather than bone marrow, blood, lung, spleen, liver and brain (Supplementary Fig. 3f). Moreover, kidney was the major target organ with the highest fungal burden with *C. albicans* infection (Fig. R2). These data indicated that the kidney was the most central target organ for neutrophil migration during *C. albicans* infection. On the other hand, we performed the microinjection of PE-labelled CD45 antibody into the tibias of mice as a cell tracker for tracing neutrophil migration and found that PD-L1 deficiency significantly increased the frequency of PE-labelled neutrophil migration from the bone marrow into kidneys of mice on Day 4 and 6 after *C. albicans* infection (Fig. 4a). Consequently, PD-L1 deficiency led to a significant decrease of neutrophil accumulation in the bone marrow and an obvious increase of neutrophil infiltration into the peripheral blood and kidney on Day 4 and 6 after *C. albicans* infection (Fig. 4b and Supplementary Fig. 4a-b). These data suggested that PD-L1 was involved in regulating neutrophil migration from the bone marrow into the peripheral blood and kidney during *C. albicans* infection.

More importantly, we suggested that PD-L1 regulated the production of CXCL1 and CXCL2 by neutrophil in an autocrine secretion manner to govern their migration from the bone marrow into the peripheral blood and kidneys of *C. albicans*-infected mice. We first found that CXCL1 and CXCL2 were prominently produced by neutrophils in the bone marrow of *C. albicans*-infected mice (Fig. 3j and Supplementary Fig. 3g), whereas, in the kidneys of infected mice, the proportion of CXCL1 and CXCL2 secreted by neutrophils was very low (Fig. 3j and Supplementary Fig. 3g). In addition, we performed the microinjection of their neutralizing antibodies against CXCL1 or CXCL2 into the tibias of wild-type and PD-L1-deficient mice. We found that blockade of either CXCL1 or CXCL2 led to a significant decrease of neutrophil accumulation in the bone marrow and an obvious increase of neutrophil infiltration into kidneys of wild-type mice on Day 4 after *C. albicans* infection, accompanied with a significant decrease of kidneys fungal burden (Fig. 4f-i and Supplementary Fig. 4e-f). However, blocking either CXCL1 or CXCL2 had no influences on neutrophil migration from the bone marrow into kidneys and fungal burden in PD-L1-deficienct mice (Fig. 4f-i and Supplementary Fig. 4e-f). Meanwhile, we found that neutrophil-specific deficiency of PD-L1 significantly impaired the production of CXCL1 and CXCL2 in the bone marrow of mice on Day 4 after *C. albicans* infection whereas no changes of CXCL1 and CXCL2 were observed in the peripheral blood and kidneys between CD274$^{fl/fl}$ and CD274$^{fl/fl}$MRP8$^{Cre/+}$ mice (Fig. 5d). However, we will explore whether some other chemokines are elevated in the PD-L1 KO kidney, thereby driving the increased PMN recruitment in our future studies.

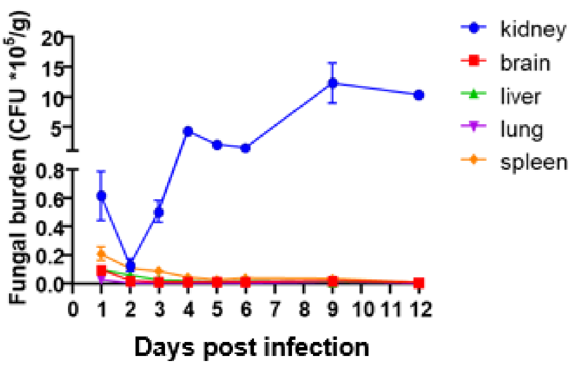

Fig R2 Fungal burden in the kidney, brain, liver, lung and spleen of wild-type mice, which were intravenously infected with 2×10^5 CFUs of *C. albicans* strain SC5314 for 12 days (n=6 for each group).

3. Ageing PMN have been shown to express PDL1. Do the authors think their PDL1+ PMN are an aged population that accumulate during fungal infection? Can they look for aging signatures in their RNAseq dataset or stain for other markers of aged PMN (e.g. CXCR4)? Aged PMN have quite different functional properties so it would be interesting to see if this poorly understood population are what is being detected in the bone marrow of infected mice.

Response: Thank you for your insightful comments. It has been well-documented that increased CXC-chemokine receptor 4 (CXCR4) expression can be observed in aged neutrophils, which is thought to help direct them back to the bone marrow, where they are then eliminated (Kolaczkowska et al. *Nat Rev Immunol. 2013, 13(3):159-75.*). However, we found that the frequency of murine neutrophils expressing CXCR4 was significantly decreased after stimulation with β-glucan-containing particle curdlan (Fig. R3), which might rule out the possibility that PD-L1$^+$ PMN are an aged population that accumulate during fungal infection.

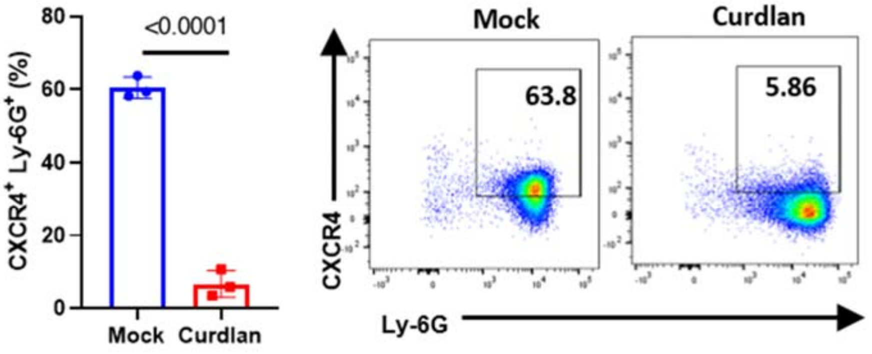

Fig R3 The percentage and flow cytometry analysis chart of CXCR4^{+}Ly-6G^{+} mu-PMNs after stimulation with curdlan (25μg/well for mu-PMNs) for 12 hours. Mean ± SD from n=3.

Minor comments:

1. Fig 3J; the PE labelling of PMN in the kidney is not very convincing. How are the gates being set? Can the authors please provide a negative control so that positive staining can be proven?

Response: Thank you for your helpful comments. We repeated these data and showed our gating strategy in Fig. 3k of our revised manuscript. In detail, we added an isotype control as a negative control and examined the frequency of PE-labelled neutrophils in the bone marrow and kidneys of naïve and *C. albicans*-infected mice, which were microinjected with PE-labelled CD45 antibody as a cell tracker entered the tibia 12 hours before sacrifice on the indicated days.

2. The western blots and FACS for JAK2/STAT3 are shown as representative data – but this is not stated. Can the authors please provide quantitative data or state how many times this experiment was done (i.e. how reproducible is the result)?

Response: Thank you for your suggestion. We performed immunoblot-based assay for examining the phosphorylation of JAK2 and STAT3, which were included in Fig. 1g, 1h, and 7a as representative data from three biologically independent samples, as described in the Methods section of Statistics and reproducibility in our revised manuscript.

3. RNA seq data needs deposited.

Response: The sequence data generated in this study have been deposited in the GEO database under the accession code PRJNA786718 (https://www.ncbi.nlm.nih.gov/bioproject/PRJNA786718) and PRJNA786748 (https://www.ncbi.nlm.nih.gov/bioproject/PRJNA786748) as described in the Methods section of Data availability in our revised manuscript.

Reviewer #2 (Remarks to the Author):

In this robust paper, Yao Yu et al show that during C. albicans infection, PD-L1 governs the mobilization of neutrophils through regulating their autocrine secretion of CXCL1 and CXCL2. In support of the above mechanism, they present that deficiency or blockade of PD-L1 significantly impairs the secretion of CXCL1 and CXCL2 in murine or human neutrophils induced by â-glucans, and subsequently suppresses neutrophil mobilization as a consequence. This is a nice, multilayered, and novel story, that extends to the "hot" topic of the role of Check point blockage and fungal immunity. Although the upregulation of the PD-1/PDL1 pathway and protective effects of PD-1 pathway inhibitors have been previously shown in murine models of invasive candidiasis/Candida sepsis, this study provides a first deep-dive mechanistic exploration of the involvement of PD-L1 in immune cell recruitment to the site of infection. Part of the potential significance of this study is its focus on the autocrine downstream effects of PD-L1. Most cancer immunology studies rather focused on the upstream regulation of PD-L1 expression and the implications of PD-L1 expression for interactions with other immune cells (especially lymphocytes), whereas PD-L1 effects in neutrophils are relatively scarcely studied. However, many unanswered questions remain regarding both the signaling cascades resulting in beta-glucan-induced PD-L1 upregulation and the specific downstream effects. It also remains unclear how PD-L1 specifically retains neutrophils in the bone marrow, as the in vitro experiments showed an effect of PD-L1 deficiency on CXCL1/2 production in both (murine) bone marrow derived neutrophils and (human) peripheral blood neutrophils. Furthermore, some critical links between the findings have not been sufficiently proven experimentally. Most significantly, in order to demonstrate that the therapeutic effect of PD-L1 against invasive candidiasis predominantly relies on the CXCL1/2-dependent retention of neutrophils in the bone marrow, the authors should use a CXCL1/2-deficient mouse strain and show that the therapeutic effect of PD-L1 significantly vanishes compared to CXCL1/2-producing mice. Finally, he biggest issue with the paper is the " timing question as assessments were mostly static, whereas these are highly dynamic processes. Studying these aspects serially and get a better feeling of the kinetics to make inferences

regarding the optimal timing of combinatorial strategies. Consider discussing those issues as a limitation.

Specific comments:

• As indicated above, it remains entirely unclear from the authors' data why the PD-L1 effect on neutrophil attraction/retention is specifically seen in the bone marrow. In fact, the authors show PD-L1-dependent Curdlan-induced CXCL1/2 secretion in vitro in neutrophils obtained from peripheral human blood. Therefore, the question arises why PD-L1 only mediates the CXCL1/2- dependent retention of neutrophils in the bone marrow as opposed to, for example, active CXCL1/2-dependent recruitment of neutrophils to the kidney or into the bloodstream? This question is neither experimentally addressed nor sufficiently discussed. Of note studies have shown that the pharmacological Blockade of the Chemokine Receptor CCR1 Protects Mice from Systemic Candidiasis (see Antimicrob Agents Chemother. 2017 Feb 23;61(3):e02365-16. doi: 10.1128/AAC.02365-16)

Response: Thank you for your comments. According to your suggestions, we performed intracellular staining to determine the CXCL1 and CXCL2-producing cells and found that CXCL1 and CXCL2 were prominently produced by neutrophils in the bone marrow of *C. albicans*-infected mice (Fig. 3j and Supplementary Fig. 3g). Whereas, in the kidneys of infected mice, the proportion of CXCL1 and CXCL2 secreted by neutrophils was very low (Fig. 3j and Supplementary Fig. 3g). Moreover, we performed the microinjection of their neutralizing antibodies against CXCL1 or CXCL2 into the tibia of wild-type and PD-L1-deficient mice. We found that blockade of either CXCL1 or CXCL2 led to a significant decrease of neutrophil accumulation in the bone marrow and an obvious increase of neutrophil infiltration into kidneys of wild-type mice on Day 4 after *C. albicans* infection, accompanied with a significant decrease of kidneys fungal burden (Fig. 4f-i and Supplementary Fig. 4e-f). However, blocking either CXCL1 or CXCL2 had no influences on neutrophil migration from the bone marrow into kidneys and fungal burden in PD-L1-deficienct mice (Fig. 4f-i and

Supplementary Fig. 4e-f). Importantly, we found that neutrophil-specific deficiency of PD-L1 significantly impaired the production of CXCL1 and CXCL2 in the bone marrow of mice on Day 4 after *C. albicans* infection whereas no changes of CXCL1 and CXCL2 were observed in the peripheral blood and kidneys between CD274$^{fl/fl}$ and CD274$^{fl/fl}$MRP8$^{Cre/+}$ mice (Fig. 5d). Consequently, neutrophil-specific deficiency of PD-L1 led to a significant decrease of neutrophil accumulation in the bone marrow and an obvious increase of neutrophil infiltration into the peripheral blood and kidneys on Day 4 and 6 after *C. albicans* infection (Fig. 5c and Supplementary Fig. 5b-c). Together, these data indicated that *C. albicans* infection induced PD-L1 expression in neutrophils to regulate their autocrine secretion of CXCL1 and CXCL2, which led to neutrophil accumulation in the bone marrow of mice.

We further examined whether the expression of the Chemokine Receptor CCR1 was regulated by PD-L1 and found that PD-L1 deficiency had no influence on CCR1 expression in murine neutrophils after challenge with β-glucan-containing particle curdlan (Fig. R4), which ruled out the possibility that PD-L1 governed the mobilization of neutrophils through regulating CCR1 expression.

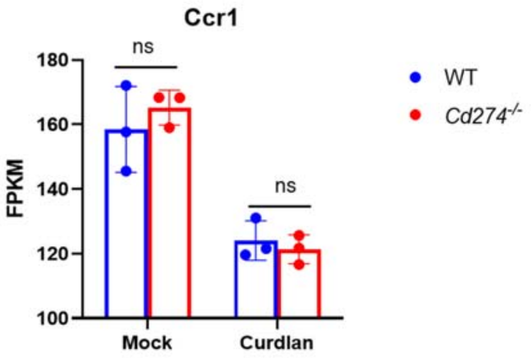

Fig R4 CCR1 enrichment in wild-type, *Clec7a*$^{-/-}$ and *Cd274*$^{-/-}$ mu-PMNs, which were stimulated with or without curdlan(25μg/well) for 4 hours.

• Lines 131-132 and Fig. 1B: Were other checkpoint/exhaustion markers upregulated as well or did CD274/PD-L1 stand out as the only/strongest checkpoint signal?

Response: Thank you for your comment. As shown in Fig. 1b, CD274 is the only

upregulated checkpoint/exhaustion marker in our present study. More importantly, either deficiency of Dectin-1 in murine neutrophils or blockade of Dectin-1 in human neutrophils significantly decreased the PD-L1-expressing frequency induced by *C. albicans* yeast and β-glucans (Fig. 1e and Supplementary Fig. 1d-e).

• Many effects shown in Fig. 1 and 2 are based on a static assessment of a single timepoint. Ideally, a more dynamic assessment to show that PD-L1 upregulation on the cell surface of neutrophils precedes the downstream effects on migration and CXCL1/2 expression would make the story line stronger.

Response: Thank you for your good suggestions. As shown in Fig. 1c, we used flow cytometry to validate that the frequency of human and murine neutrophils expressing PD-L1 was significantly increased after stimulation with heat-inactivated *C. albicans* yeast in a dose-dependent manner. We also showed that β-glucan stimulation significantly increased PD-L1 expression in human and murine neutrophils in a time-dependent manner (Supplementary Fig. 1c and Fig. R5). Direct *ex vivo* assay showed that β-glucan stimulation induced the secretion of CXCL1 and CXCL2 to the peak in murine neutrophils for 4h, 6h and 8h (Supplementary Fig. 2c).

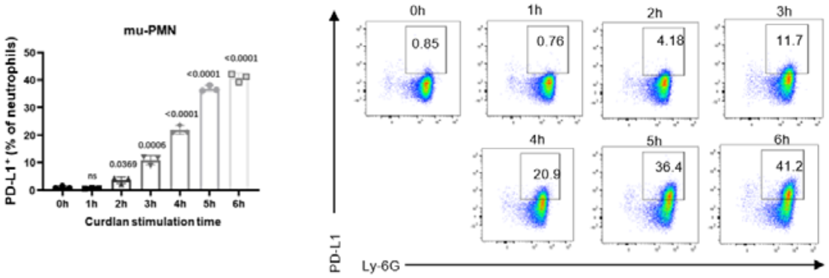

Fig R5 The percentage and flow cytometry analysis chart of PD-L1$^+$Ly-6G$^+$ mu-PMNs after stimulation with curdlan (25μg/well for mu-PMNs) for the indicated time. Mean ± SEM from n=3.

• Multiple distinct downstream signaling pathways of Dectin-1 have been described and it remains unclear from the data presented how exactly Dectin-1 signaling leads to PD-

L1 induction via JAK2 and STAT3 phosphorylation. The described Dectin-1 signaling pathways either rely on Syk, NF-kB, and/or PI3K (see a recent detailed review, PMID: 35237264). The authors show data (knockout mice or inhibitors) suggesting that all 3 proteins are not essential for PD-L1 induction. Therefore, PD-L1 induction either relies on multiple, redundant pathways, or a previously unknown pathway, but this is overall quite unclear from the authors' data (and not further discussed). It is also possible that indirect effects, e.g., through an interferon signaling loop, might be involved.

Response: According to your suggestion, we found that the inhibition of STAT3 activation with its specific inhibitor stattic, rather than bortezomib (NF-κB inhibitors) and wortmannin (PI3K-Akt inhibitors), most significantly reduced the frequency of murine neutrophils expressing PD-L1 induced by β-glucans (Supplementary Fig. 1i). Moreover, immunoblot-based assay showed that β-glucans stimulation significantly promoted the phosphorylation of JAK2 and STAT3 in murine neutrophils (Fig. 1g). Furthermore, Dectin-1 deficiency impaired β-glucan-induced phosphorylation of JAK2 and STAT3 in murine neutrophils (Fig. 1h). However, stattic treatment further decreased β-glucan-induced PD-L1-expressing frequency in Dectin-1-deficient murine neutrophils (Fig. 1i and Supplementary Fig. 1j), indicating that PD-L1 expression was mainly governed by JAK2/STAT3 pathway, which was partially regulated by Dectin-1. We further found that treatment with ACT001, which can block PD-L1 expression by inhibiting the phosphorylation of STAT3 in glioblastoma, and could inhibit β-glucan-induced phosphorylation of JAK2 and STAT3 in murine neutrophils (Fig. 7a). Moreover, ACT001 treatment significantly inhibited β-glucan-induced expression of PD-L1 in murine and human neutrophils in a dose-dependent manner (Fig. 7b). However, ACT001 treatment further decreased β-glucan or *C. albicans* yeast-induced PD-L1-expressing frequency in Dectin-1-deficient murine neutrophils (Fig. 7c and Supplementary Fig. 7a), which further confirmed that PD-L1 expression was mainly governed by STAT3 pathway, which was partially regulated by Dectin-1.

We further found that blocking IFN-γ with its specific antibody had no

influence on β-glucan-induced frequency of murine neutrophils expressing PD-L1 (Fig. R6), which ruled out the possibility that interferon signaling is involved in regulating β-glucan-induced expression of PD-L1 in neutrophils.

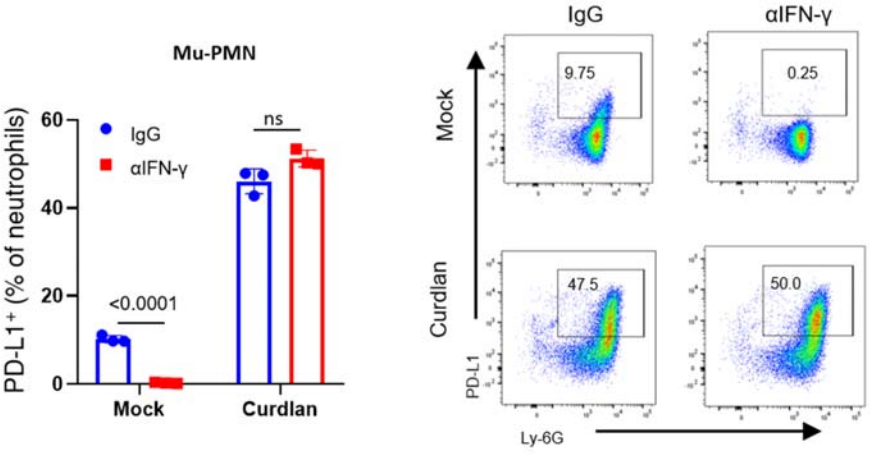

Fig R6 The percentage and flow cytometry analysis chart of PD-L1$^+$Ly-6G$^+$ mu-PMNs after treatment with anti- IFN-γ, which were co-stimulated with curdlan (25μg/well for mu-PMNs) for 12 hours.

• Fig. 2H: This experiment does not necessarily point to an involvement of PD-L1. Ivermectin blocks nuclear import in an unspecific manner and would also block other potentially critical nuclear factors involved in Dectin-1 signaling, e.g., IRF5 or NFAT.

Response: It has been shown that nuclear PD-L1 can couple to transcription factor Sp1 to regulate the synthesis of Gas6 mRNA (*Cell Death Differ. 2021, 28(4):1284-1300*). We found that the inhibition of Sp1 activation by plicamycin further decreased β-glucan-induced production of CXCL1 and CXCL2 in PD-L1-deficient murine neutrophils (Fig. 2i), indicating that CXCL1 and CXCL2 production was mainly controlled by Sp1 pathway, which might be partially regulated by PD-L1. Moreover, treatment with nuclear factor of activated T cells (NFAT) activation inhibitor in wild-type neutrophils resulted in comparable reductions in the amount of CXCL1 and CXCL2 compared with untreated PD-L1-deficient neutrophils, but this treatment further decreased CXCL1 and CXCL2 amount in PD-L1-deficient neutrophils (Supplementary Fig. 2f). These

data implied that CXCL1 and CXCL2 production was also controlled by NFAT pathway, which might be partially regulated by PD-L1. However, inhibition of interferon regulatory factor 5 (IRF5) activation by N5-1 had no influences on β-glucan-induced production of CXCL1 and CXCL2 in either wild-type or PD-L1-deficient neutrophils (Supplementary Fig. 2g), implying that IRF5 was not involved in the regulation of CXCL1 and CXCL2 production.

• Does PD-L1 expression affect other (direct) neutrophil effector functions that are relevant for fungal clearance, e.g., oxidative burst or NET formation?

Response: Thank you for your valuable suggestion. We found that PD-L1 deficiency affected neither neutrophil killing against *C. albicans* nor β-glucan-induced ROS production and NET formation (Fig. R7).

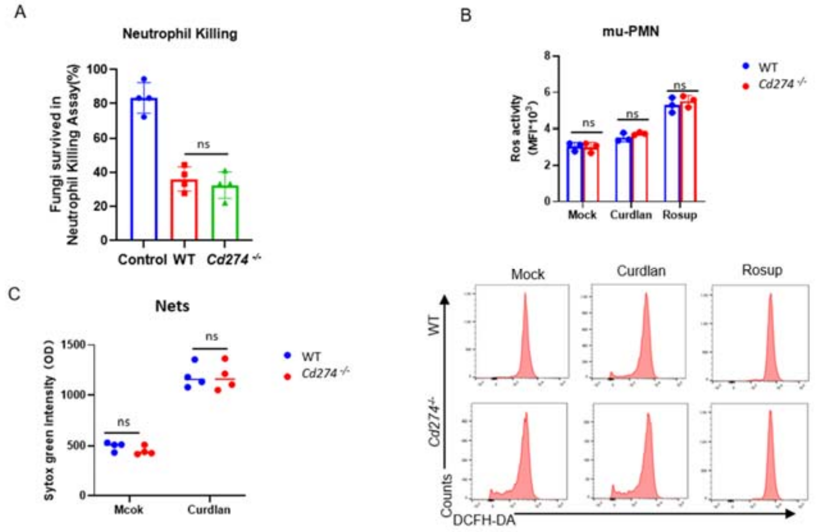

Fig R7 (A) Fungal killing results of wild-type and *Cd274*$^{-/-}$ mu-PMNs. **(B)** ROS production and flow cytometry analysis chart of wild-type and *Cd274*$^{-/-}$ mu-PMNs, which were stimulated with curdlan(25μg/well) for 4 hours, Rosup: positive control. **(C)** NET formation of wild-type and *Cd274*$^{-/-}$ mu-PMNs, which were stimulated with curdlan(25μg/well) for 8 hours. Mean ± SD from n=3.

• The focus on only two compartments (kidney and bone marrow) is an over-simplification of the complex regulation of neutrophil recruitment. For instance, others have previously shown that PD-L1-expressing neutrophils can transiently arrest in the

lung vasculature, possibly contributing to injury-induced infection susceptibility (PMID: 33674305). The spleen might be another highly pertinent repertoire. Therefore, more dynamic studies and assessment of the accumulation of labelled neutrophils in additional organs will be needed to better understand the complex dynamics.

Response: Regarding to your good suggestion, we adoptively transferred GFP$^+$ neutrophils into *C. albicans*-infected mice, and found that kidney was the organ most infiltrated by GFP$^+$ neutrophils, rather than bone marrow, blood, lung, spleen, liver and brain (Supplementary Fig. 3f). Moreover, kidney was the major target organ with the highest fungal burden with *C. albicans* infection (Fig. R2). These data indicated that the kidney was the most central target organ for neutrophil migration during *C. albicans* infection.

More importantly, we aimed to clarify that *C. albicans* infection induced PD-L1 expression in neutrophils, which inhibited neutrophil release from the bone marrow into the kidney to aggravate *C. albicans* infection. For this purpose, we performed the microinjection of PE-labelled CD45 antibody into the tibias of wild-type, PD-L1 KO, CD274$^{fl/fl}$ and CD274$^{fl/fl}$MRP8$^{Cre/+}$ mice as a cell tracker for tracing neutrophil migration and found that both PD-L1 deficiency and neutrophil-specific deficiency of PD-L1 significantly increased the frequency of PE-labelled neutrophil migration from the bone marrow into kidneys of mice on Day 4 and 6 after *C. albicans* infection (Fig. 4a and 5b). Consequently, both PD-L1 deficiency and neutrophil-specific deficiency of PD-L1 led to a significant decrease of neutrophil accumulation in the bone marrow and an obvious increase of neutrophil infiltration into the peripheral blood and kidney on Day 4 and 6 after *C. albicans* infection (Fig. 4b, 5c and Supplementary Fig. 4a-b, 5b-c). These data suggested that PD-L1 was involved in regulating neutrophil migration from the bone marrow into the peripheral blood and kidney during *C. albicans* infection.

Consider discussing as a limitation

• The conclusions in lines 255-257 and 303-304 do not fully align with the raw data.

Response: Thank you for your suggestion. We have revised the sentences in lines

255-257 as following: "Thus, these data implied that *C. albicans* infection induced neutrophil accumulation in the bone marrow and inhibited neutrophil migration into the circulation.", and revised the sentence in lines 303-304 as following: "Thus, these data confirmed that *C. albicans* infection induced PD-L1 expression in neutrophils, which involved in regulating neutrophil release from the bone marrow into the kidney."

Fig. 3I-J suggest that neutrophils are initially not retained in the bone marrow but recruited to the infected tissue despite PD-L1 expression. From the data presented, it rather seems that PD-L1 might impact the secondary re-distribution between peripheral tissues and bone marrow (between days 2 and 4). This would be more difficult to align with the in vitro studies presented. Unfortunately, dynamic studies with multiple time points are lacking in the experiments with PD-L1 deficient mice and the PD-L1 inhibitor (Fig. 4 and 5). Therefore, it is difficult to fully appreciate the role of PD-L1 in the initial and secondary neutrophil distribution kinetics.

Response: Thank you for your suggestions. As described in the above response, our present study aimed to clarify that *C. albicans* infection induced PD-L1 expression in neutrophils, which inhibited neutrophil release from the bone marrow into the kidney to aggravate *C. albicans* infection. For this purpose, we performed the microinjection of PE-labelled CD45 antibody into the tibias of wild-type, PD-L1 KO, CD274$^{fl/fl}$ and CD274$^{fl/fl}$MRP8$^{Cre/+}$ mice as a cell tracker for tracing neutrophil migration and found that both PD-L1 deficiency and neutrophil-specific deficiency of PD-L1 significantly increased the frequency of PE-labelled neutrophil migration from the bone marrow into kidneys of mice on Day 4 and 6 after *C. albicans* infection (Fig. 4a and 5b). Meanwhile, both PD-L1 deficiency and neutrophil-specific deficiency of PD-L1 led to a significant decrease of neutrophil accumulation in the bone marrow and an obvious increase of neutrophil infiltration into the peripheral blood and kidney on Day 4 and 6 after *C. albicans* infection (Fig. 4b, 5c and Supplementary Fig. 4a-b, 5b-c). These data suggested that PD-L1 was involved in regulating neutrophil migration from the

bone marrow into the peripheral blood and kidney during *C. albicans* infection. However, we agree with you that it is difficult to distinguish the role of PD-L1 in the initial and secondary neutrophil distribution kinetics, which will be explored in our future studies.

• From the data presented, it ultimately remains unclear that the therapeutic effect of PD-L1 against invasive candidiasis predominantly relies on CXCL1/2-dependent neutrophil migration. It is noteworthy that the mortality advantage of anti-PD-L1 is only seen after 5-10 days, whereas the effects on neutrophil migration are seen earlier. Therefore, it remains unclear whether the therapeutic benefit relies on a) a delayed protective effect of increased neutrophil accumulation in the kidneys and/or b) additional independent mechanisms such as improved lymphocyte exhaustion. The authors could potentially add other (earlier) surrogates of protection, e.g. morbidity scores after 2 and 4 days or weight curves. These surrogates of protection usually precede the survival benefit. Furthermore, to further support a direct role of CXCL1/2 in anti-PD-L1-mediated protection, the authors should use a CXCL1/2-deficient mouse strain and show that the therapeutic effect of PD-L1 significantly vanishes compared to CXCL1/2-producing mice.

Response: Thank you for your good suggestion. Unfortunately, in our present study, CXCL1/2-deficient mice are not available for determining whether the therapeutic effect of PD-L1 would be significantly vanish compared to CXCL1/2-producing mice. However, we performed the microinjection of neutralizing antibodies against CXCL1 or CXCL2 into the tibias of wild-type and PD-L1-deficient mice. We found that blockade of either CXCL1 or CXCL2 led to a significant decrease of neutrophil accumulation in the bone marrow and an obvious increase of neutrophil infiltration into kidneys of wild-type mice on Day 4 after *C. albicans* infection, accompanied with a significant decrease of kidney fungal burden (Fig. 4f-i and Supplementary Fig. 4e-f). However, blocking either CXCL1 or CXCL2 had no influences on neutrophil migration from the bone marrow into kidneys and fungal burden in PD-L1-deficienct mice (Fig. 4f-i and

Supplementary Fig. 4e-f). These data indicated that PD-L1 regulated the production of CXCL1 and CXCL2 in neutrophils to govern their migration from the bone marrow into kidneys of *C. albicans*-infected mice.

General comments

• The in vivo studies are mostly based on small numbers of mice, performed with a single Candida isolate. This should be acknowledged as a limitation.

Response: According to your suggestion, we used another standard *C. albicans* strain BWP17 to intravenously infect wild-type and PD-L1-deficient mice (2×10^5 CFUs/mouse, n=5) and found that PD-L1 deficiency led to a significant decrease of neutrophil accumulation in the bone marrow and an obvious increase of neutrophil infiltration into kidney on Day 4 after infection with BWP17, accompanied with a significant decrease of fungal burden in the kidneys of PD-L1-deficient mice (Fig. R8).

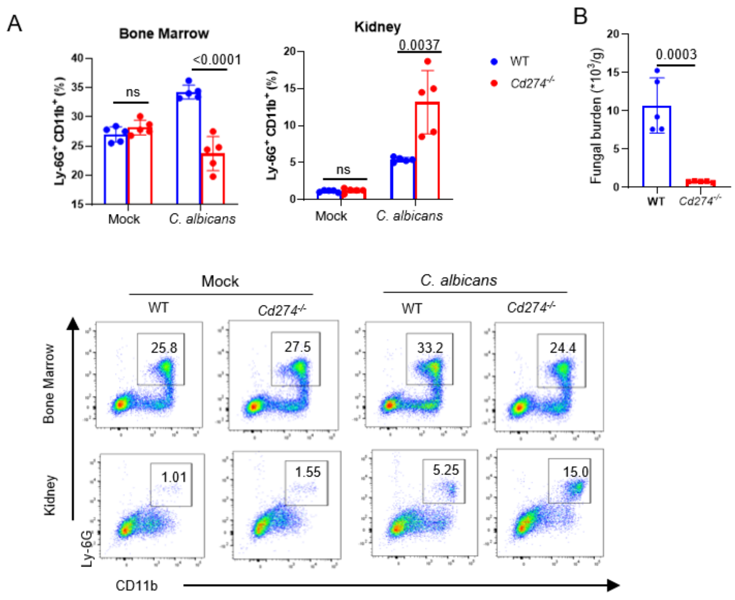

Fig R8 The percentage and flow cytometry analysis chart of neutrophils in bone marrow and kidney **(A),** and fungal burden in the kidney **(B)** of wild-type and *Cd274*$^{-/-}$ mice, which were intravenously infected with 2×10^5 CFUs of *C. albicans* strain BWP17 for 4 days (n=5 for each group).

• Several of the figure legends state that “representative dot plots from 3 independent

experiments" are shown. Does this mean that multiple replicate experiments were performed but only one is shown? If so, this should be changed by showing all data in order to provide a sense of reproducibility of the results in independent experiments with cells from different donors/mice.

Response: Thank you for your suggestion. We have revised the description of data reproducibility as following: "Data shown are representative of three biological replicates".

• Avoid the overuse of "dramatic" to describe differences between experimental cohorts/conditions. Instead, the authors could use "significant" (if applicable) or refer to the actual effect size (e.g., a 10-fold increase in…).

Response: We have revised our manuscript according to your suggestion.

• Although prior studies on PD-1/PD-L1 pathway blockade in fungal infection models have been cited in the Introduction, the authors should refer to these studies in the Discussion and put their results into a broader context, e.g., by discussing additional mechanisms likely involved in checkpoint inhibitor-mediated protection against fungal infections (e.g., reduced lymphocyte exhaustion and improved maturation and fungicidal activity of mononuclear phagocytes).

Response: Thank you for your suggestion. We have discussed this content in Discussion section of our revised manuscript as following: "Coincidentally, recent studies have shown that, PD-L1 expression in DCs induced by α-(1,3)-glucan from *Aspergillus fumigatus* through activating Wnt/β-catenin pathway promotes regulatory T-cell (Treg) polarization, suggesting that PD-L1 in DCs negatively regulates α-(1,3)-glucan-induced protective immune responses against *A. fumigatus* infection (*mBio, 2021, 12, e0282421; J Infect Dis, 2017, 216, 1281-1294*). Moreover, it has been further reported that, PD-L1 expression in macrophages induced by *Cryptococcus neoformans* infection suppresses macrophage activation, and PD-L1 expression in T cells induced by *C. albicans* infection can promote T cell exhaustion (*J Surg Res, 2017, 208, 33-39; J Cell Biochem, 2018, 119, 3044-*

***3057*). It remains unclear whether PD-L1 expression in neutrophils induced by β-glucans can affect Treg polarization, macrophage activation or T cell exhaustion during *C. albicans* infection, which would be further explored in our future studies.”**

• Some of the panels are blurry (e.g., Fig. 1G, 2J).

Response: We have updated these blurry panels in our revised manuscript.

Reviewer #3 (Remarks to the Author):

The authors report that PD-L1 regulate anti-fungal response by regulating the neutrophil migration. Other recent reports have demonstrated that PD-L1 can be induced in innate cells like dendritic cells upon stimulation with glucans or fungi like Aspergillus fumigatus. Also, PD-L1 affects the T cell responses as well (PMID: 34781737, 28968869). Unfortunate that authors have not given due credit to those reports. PD-L1 thus can regulate the anti-fungal immunity by several mechanisms and not just by regulating the neutrophil migration.

Response: Thank you for your suggestion. We have discussed this content in Discussion section of our revised manuscript as following: "Coincidentally, recent studies have shown that, PD-L1 expression in DCs induced by α-(1,3)-glucan from *Aspergillus fumigatus* through activating Wnt/β-catenin pathway promotes regulatory T-cell (Treg) polarization, suggesting that PD-L1 in DCs negatively regulates α-(1,3)-glucan-induced protective immune responses against *A. fumigatus* infection (*mBio, 2021, 12, e0282421; J Infect Dis, 2017, 216, 1281-1294*). Moreover, it has been further reported that, PD-L1 expression in macrophages induced by *Cryptococcus neoformans* infection suppresses macrophage activation, and PD-L1 expression in T cells induced by *C. albicans* infection can promote T cell exhaustion (*J Surg Res, 2017, 208, 33-39; J Cell Biochem, 2018, 119, 3044-3057*). It remains unclear whether PD-L1 expression in neutrophils induced by β-glucans can affect Treg polarization, macrophage activation or T cell exhaustion during *C. albicans* infection, which would be further explored in our future studies."

Fig 1:

CLEC7A deficient neutrophils though showed reduced PD-L1 upon stimulation with curdlan; PD-L1 expression is still high. Also, the human data of using anti-Dectin-1 blocking antibodies is not convincing Needed to validate the results on PD-L1 expression with live candida. The lack of involvement of Syk or CARD9 need further probing on the pathway implicated. Otherwise, the data are not complete.

Response: We thank the reviewer for important suggestion. Firstly, we observed

that deficiency of Dectin-1 in murine neutrophils significantly decreased the PD-L1-expressing frequency induced by *C. albicans* yeast (Fig. 1e and Supplementary Fig. 1d-e), rather than *C. albicans* hyphae and live *C. albicans* (Fig.R9 A-B). Of note, live neutrophils significantly decreased after co-culture with live *C. albicans* (Fig.R9 A). Regarding to your concerns that human data using anti-Dectin-1 blocking antibodies, we repeated these data with more tested time (Fig. 1e and Supplementary Fig. 1d-e). Furthermore，to explore the involvement of Syk or CARD9，we confirmed that either the deficiency of Syk or CARD9 had no influences on the β-glucan-induced PD-L1-expressing frequency in murine neutrophils (Fig. 1f and Supplementary Fig. 1f). We also found that neither inhibition of NF-κB activation by bortezomib nor inhibition of Syk activation by Piceatannol decreased the β-glucan-induced PD-L1-expressing frequency (Supplementary Fig. 1i and Fig.R9 C).

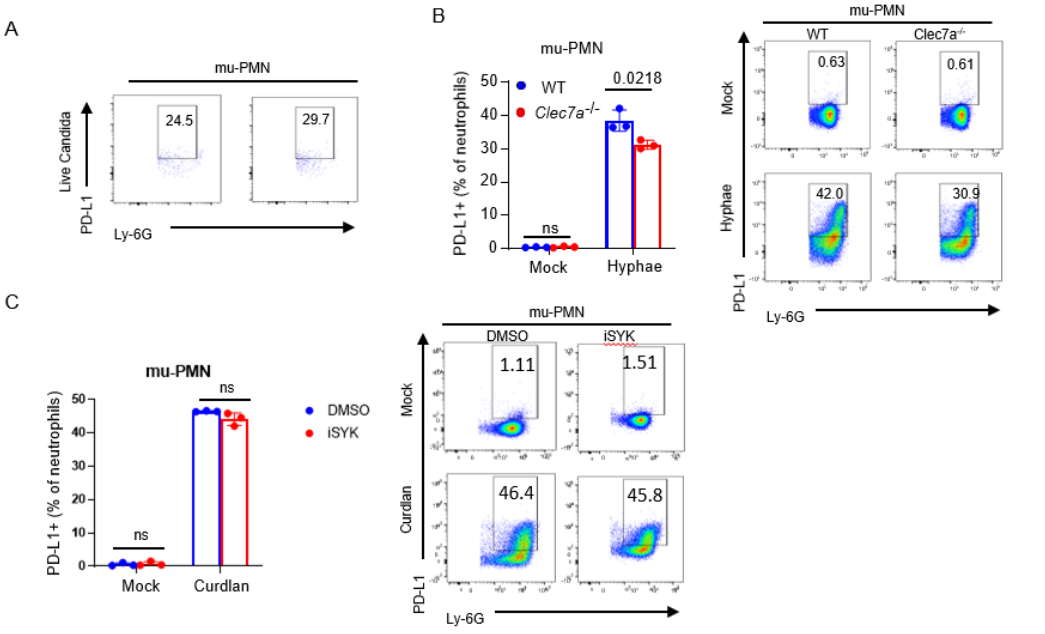

Fig R9 (A) The flow cytometry analysis chart of PD-L1^{+}Ly-6G^{+}mu-PMNs after stimulation with *live C. albicans* (MOI=0.01) for 12 hours. Mean ± SD from n=3. (**B**) The percentage and flow cytometry analysis chart of PD-L1^{+}Ly-6G^{+}mu-PMNs after stimulation with UV-inactivated *C. albicans hyphae* (MOI=0.1) for 12 hours. Mean ± SD from n=3. (**C**) The percentage and flow cytometry analysis chart of PD-L1^{+}Ly-6G^{+}mu-PMNs after stimulation with Curdlan combined with inhibitor Piceatannol (10μM) for 12 hours. Mean ± SD from n=3.

Fig 1J: pJAK2 spectra are not convincing

Response: We performed immunoblot-based assay for examining the phosphorylation of JAK2 and STAT3, which were included in Fig. 1g and 1h as representative data from three biological independent samples in our revised manuscript.

Fig 1G: Give the precise pathway implicated by data mining of the RNA seq data instead of listing which are upregulated

Response: We have revised the description as following: "Kyoto Encyclopedia of Genes and Genomes (KEGG) analysis revealed that CLR pathway, Janus kinase (JAK) and signal transducer and activator of transcription (STAT) pathway, NF-κB pathway and PI3K-Akt pathway might be involved in regulating *C. albicans* yeast and β-glucan-induced expression of PD-L1 (Supplementary Fig. 1h)." in our revised manuscript.

Fig 2: The effect of PD-L1 deficiency on CXCL1 secretion of murine neutrophils and conclusions made are not convincing. Why PD-L1 deficiency should lead to downregulation of genes including CXCL1 and CXCL2 in neutrophils upon stimulation with b-glucan? It is not a receptor for b-glucan. There are no ligands present in the experiments which signal through PD-L1. Showing that PD-L1 translocate into the nucleus does not prove that it affects the production of CXCL1 and CXCL2 in neutrophils. Need to show whether PD-L1 binds the promoter region of CXCL1 and CXCL2 genes. What is the effect of PD-L1 or Dectin-1 deficiency on the expression of CXCR2 on the neutrophils?

Response: Thank you for your good suggestions. It has been shown that nuclear PD-L1 can couple to transcription factor Sp1 to regulate the synthesis of Gas6 mRNA (*Cell Death Differ. 2021, 28(4):1284-1300*). We found that the inhibition of Sp1 activation by plicamycin further decreased β-glucan-induced production of CXCL1 and CXCL2 in PD-L1-deficient murine neutrophils (Fig. 2i), indicating that CXCL1 and CXCL2 production was mainly controlled by Sp1 pathway,

which might be partially regulated by PD-L1. Moreover, treatment with nuclear factor of activated T cells (NFAT) activation inhibitor in wild-type neutrophils resulted in comparable reductions in the amount of CXCL1 and CXCL2 compared with untreated PD-L1-deficient neutrophils, but this treatment further decreased CXCL1 and CXCL2 amount in PD-L1-deficient neutrophils (Supplementary Fig. 2f). These data implied that CXCL1 and CXCL2 production was also controlled by NFAT pathway, which might be partially regulated by PD-L1. However, inhibition of interferon regulatory factor 5 (IRF5) activation by N5-1 had no influences on β-glucan-induced production of CXCL1 and CXCL2 in either wild-type or PD-L1-deficient neutrophils (Supplementary Fig. 2g), implying that IRF5 was not involved in the regulation of CXCL1 and CXCL2 production.

On the other hand, we found that either PD-L1 or Dectin-1 deficiency had no influence on β-glucan-induced frequency of murine neutrophils expressing CXCR2 (Fig. R10), which ruled out the possibility that Dectin-1 and PD-L1 may be involved in regulating β-glucan-induced expression of CXCR2 in neutrophils.

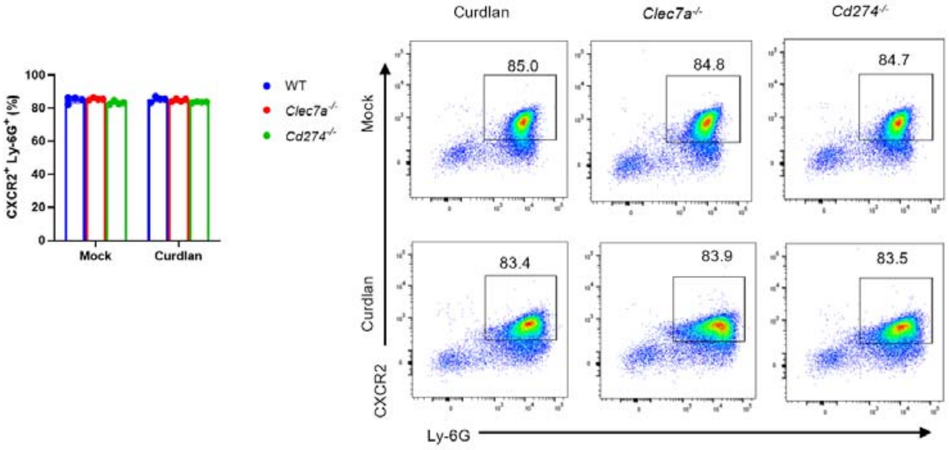

Fig R10 The percentage and flow cytometry analysis chart of CXCR2 frequency in wild-type, *Clec7a*$^{-/-}$ and *Cd274*$^{-/-}$ mu-PMNs after stimulation with curdlan (25μg/well for mu-PMNs) for 12 hours. Mean ± SD from n=3.

Fig 3: Dectin-1 and PD-L1 are expressed on many innate cells and the use of KO mice not specific for the neutrophils does not validate the results. The effects are also possibly due to the other immune cells rather than the direct effect on the neutrophils.

What is the repercussion of migration of other innate cells like monocytes, macrophages, dendritic cells to the bone marrow in all these different conditions and experiments? Also CXCL1/2 are produced by other cells as well.

Response: To confirm the direct role of PD-L1 on neutrophil migration, we generated mice with conditional Cre/LoxP deletion of PD-L1 ($CD274^{fl/fl}MRP8^{Cre/+}$) in neutrophils by breeding $CD274^{fl/fl}$ mice to $MRP8^{Cre/+}$ mice as shown in Fig. 5 of our revised manuscript. We confirmed that PD-L1 was deficient in $Ly6G^{+}$ neutrophils in the bone marrow and kidneys of infected mice with *C. albicans* (Fig. 5a and Supplementary Fig. 5a).

On the other hand, we found that CXCL1 and CXCL2 were prominently produced by neutrophils in the bone marrow of *C. albicans*-infected mice (Fig. 3j and Supplementary Fig. 3g). Whereas, in the kidneys of infected mice, the proportion of CXCL1 and CXCL2 secreted by neutrophils was very low (Fig. 3j and Supplementary Fig. 3g).

Fig 4A-D: What is the mechanism and pathway by which PD-L1 promotes neutrophil migration to the kidneys from the bone marrow? Of note at Day 2, no influence of PD-L1 for the neutrophil migration to the kidney and effect is visible on day 4 ? Why?

Response: We proposed that PD-L1 regulated the production of CXCL1 and CXCL2 by neutrophil in an autocrine secretion manner to govern their migration from the bone marrow into the peripheral blood and kidney of *C. albicans*-infected mice. For this purpose, we performed the microinjection of their neutralizing antibodies against CXCL1 or CXCL2 into the tibias of wild-type and PD-L1-deficient mice. We found that blockade of either CXCL1 or CXCL2 led to a significant decrease of neutrophil accumulation in the bone marrow and an obvious increase of neutrophil infiltration into kidneys of wild-type mice on Day 4 after *C. albicans* infection, accompanied with a significant decrease of kidney fungal burden (Fig. 4f-I and Supplementary Fig. 4e-f). However, blocking either CXCL1 or CXCL2 had no influences on neutrophil migration from the bone marrow into kidney and fungal burden in PD-L1-deficienct mice (Fig. 4f-I and

Supplementary Fig. 4e-f). Meanwhile, we found that neutrophil-specific deficiency of PD-L1 significantly impaired the production of CXCL1 and CXCL2 in the bone marrow of mice on Day 4 after *C. albicans* infection whereas no changes of CXCL1 and CXCL2 were observed in the peripheral blood and kidney between CD274$^{fl/fl}$ and CD274$^{fl/fl}$MRP8$^{Cre/+}$ mice (Fig. 5d). Consequently, neutrophil-specific deficiency of PD-L1 led to a significant decrease of neutrophil accumulation in the bone marrow and an obvious increase of neutrophil infiltration into the peripheral blood and kidney on Day 4 and 6 after *C. albicans* infection (Fig. 5c and Supplementary Fig. 5b-c). Together, these data indicated that *C. albicans* infection induced PD-L1 expression in neutrophils to regulate their autocrine secretion of CXCL1 and CXCL2, which led to neutrophil accumulation in the bone marrow of mice.

Fig 4J: what is the effect of neutrophil specific KO of PD-L1 on the survival of mice?

Response: According to your suggestion, we found that neutrophil-specific deficiency of PD-L1 led to higher survival and lower fungal burden in the kidneys of mice than those in CD274$^{fl/fl}$ mice after infection with *C. albicans* as shown in Fig. 5e of our revised manuscript.

Fig 5: The effect of blockade of other innate cells PD-L1 cannot be ruled out

Response: To confirm the direct role of PD-L1 on neutrophil migration, we generated mice with conditional Cre/LoxP deletion of PD-L1 (CD274$^{fl/fl}$MRP8$^{Cre/+}$) in neutrophils by breeding CD274$^{fl/fl}$ mice to MRP8$^{Cre/+}$ mice as shown in Fig. 5 of our revised manuscript.

Fig 6A: pJAK2 data are not convincing

Response: We performed immunoblot-based assay for examining the phosphorylation of JAK2 and STAT3, which were included in Fig. 7a as representative data from three biological independent samples in our revised manuscript.

REVIEWERS' COMMENTS

Reviewer #1 (Remarks to the Author):

I think the revisions made to the paper have improved the data. The new gating strategies are an important addition and the tracking data are now more convincing. I like the inclusion of extra time points in the in vivo experiments as I think this helps show complexities of the dynamic antifungal response.

I think it would still be better to show pooled data rather than representative data if possible (as per one of the other reviewer's suggestions).

Overall, it's a good paper - still a lot of outstanding questions but I think it will be well cited and could stimulate new research in an area that is very poorly understood. The paper does a good job of highlighting how little we understand about dynamics of neutrophil efflux from bone marrow to different organs during infection.

Reviewer #2 (Remarks to the Author):

The authors did a comprehensive and extensive effort to address all the reviewers comments and their story is much more complete.
in regards to the discordant results in regards to other litterature (Lionakis et al. JCI 2011, could that be, in addition to inocula, the results of difference in strain and underlying genetic background of the mice? please discuss
I have no other suggestions, other than consider creating as the last figure a conceptual model that summarizes their findings.

Reviewer #3 (Remarks to the Author):

Thank you for addressing all my concerns.

POINT BY POINT REPLY TO THE REVIEWERS' COMMENTS (reproduced verbatim)

Reviewer #1 (Remarks to the Author):

I think the revisions made to the paper have improved the data. The new gating strategies are an important addition and the tracking data are now more convincing. I like the inclusion of extra time points in the in vivo experiments as I think this helps show complexities of the dynamic antifungal response.

Response: We appreciate your positive comment.

I think it would still be better to show pooled data rather than representative data if possible (as per one of the other reviewer's suggestions).

Response: Thank you for your suggestion. Due to data bias obtained from separate batch of cells and mice, it is difficulty to perform statistical analysis using pooled data. However, the difference between groups in every batch of experiments is reproducible. Thus, we show representative data in our manuscript.

Overall, it's a good paper - still a lot of outstanding questions but I think it will be well cited and could stimulate new research in an area that is very poorly understood. The paper does a good job of highlighting how little we understand about dynamics of neutrophil efflux from bone marrow to different organs during infection.

Response: Thank you for your positive comment.

Reviewer #2 (Remarks to the Author):

The authors did a comprehensive and extensive effort to address all the reviewers comments and their story is much more complete. In regards to the discordant results in regards to other litterature (Lionakis et al. JCI 2011, could that be, in addition to inocula, the results of difference in strain and underlying genetic background of the mice? please discuss

Response: Thank you for your suggestion. According to your suggestion, we carefully read this article (Lionakis et al. JCI 2011) and found that the same *C. albicans* strain (SC5314) and the same genetic background mice (C57BL/6) were used by us and Lionakis et al. Thus, we suggest that inoculation amount of *C. albicans* have great influences on the accumulation of neutrophils in kidney.

I have no other suggestions, other than consider creating as the last figure a conceptual model that summarizes their findings.

Response: According to your suggestion, we add a conceptual model as a featured image that summarizes our findings.

Reviewer #3 (Remarks to the Author):

Thank you for addressing all my concerns.

Response: We are glad to know that Reviewer #3 had no further concerns and were satisfied with our efforts to address all questions.
